# Factors Influencing the Activity of Nanozymes in the Cleavage of an RNA Model Substrate

**DOI:** 10.3390/molecules24152814

**Published:** 2019-08-01

**Authors:** Joanna Czescik, Susanna Zamolo, Tamis Darbre, Fabrizio Mancin, Paolo Scrimin

**Affiliations:** 1 University of Padova, Department of Chemical Sciences, via Marzolo, 1 35131 Padova, Italy; 2 University of Bern, Department of Chemistry and Biochemistry, Freiestrasse 3, CH-3012 Bern, Switzerland

**Keywords:** gold nanoparticles, nanozymes, phosphate cleavage, Zn(II) catalysis, guanidinium catalysis

## Abstract

A series of 2-nm gold nanoparticles passivated with different thiols all featuring at least one triazacyclonanone-Zn(II) complex and different flanking units (a second Zn(II) complex, a triethyleneoxymethyl derivative or a guanidinium of arginine of a peptide) were prepared and studied for their efficiency in the cleavage of the RNA-model substrate 2-hydroxypropyl-*p*-nitrophenyl phosphate. The source of catalysis for each of them was elucidated from the kinetic analysis (Michaelis–Menten profiles, pH dependence and kinetic isotope effect). The data indicated that two different mechanisms were operative: One involving two Zn(II) complexes and the other one involving a single Zn(II) complex and a flanking guanidinium cation. The mechanism based on a dinuclear catalytic site appeared more efficient than the one based on the cooperativity between a metal complex and a guanidinium.

## 1. Introduction

The cleavage of the phosphate ester bond constitutes one of the most challenging endeavors for a scientist [1,2,3]. The spontaneous hydrolysis of one important example, the phosphate bond connecting the nucleobases of DNA, requires, at physiological pH, billions of years to occur [4]. Yet, nucleases, the enzymes in charge of achieving this task, perform the reaction in a matter of a few seconds with accelerations up 10^16^-fold. These enzymes constitute a notable example of how cooperativity between several units present in the catalytic site may result in exceptional catalytic advantages. The most notable is that between metal centers [5,6], typically present in these proteins [7]. We first reported, almost fifteen years ago, that gold nanoparticles (AuNPs) passivated with a monolayer containing 1,4,7-triazacyclonane complexes with Zn(II) (TACN-Zn) are excellent, enzyme-like catalysts for the cleavage of the phosphate bond of the RNA-model substrate 2-hydroxypropyl-*p*-nitrophenyl phosphate (HPNP) [8]. For this reason, we coined the term “nanozymes” for these systems. Later, we showed that also oligonucleotides could be efficiently and selectively cleaved [9,10]. Other laboratories have shown the advantage of using similar nanostructures for the efficient cleavage of phosphate diesters, as well [11,12,13,14,15,16,17]. The key points at the basis of the outstanding catalytic performance of AuNPs-based nanozymes are constituted by their multivalency and the associated cooperativity between at least two metal ions in performing the catalytic process. Chin [18] has dissected the several facets of the cooperativity between two metal centers in the cleavage of a phosphate bond concluding that the most important role is played by intramolecular nucleophile activation, followed by leaving group activation and lastly Lewis acid activation (up to 10^8^, 10^6^ and 10^2^-fold acceleration, respectively). These estimates have been recently revised by Mancin [3] who estimated a larger contribution for leaving group departure assistance (up to 10^6^-fold) and a lower one for nucleophile activation (10^2^-fold).

As demonstrated by the results obtained with simple dinuclear catalysts, the cooperativity between the metal ions is not the only factor affecting reactivity. A key role is also played by the solvent. By using a less-solvating solvent than water as an alcohol, Brown has shown that the rate acceleration due to a dinuclear catalyst can be greatly improved [19]. We have shown that the polarity of the organic monolayer surrounding the gold nanoparticles plays, analogously, a very important role [20]. The reaction occurs faster in a less polar environment in line with the observation mentioned above that a major contribution to metal ion catalysis derives from the activation of the nucleophile. As it is well known and has been proven with phase transfer catalysis, a desolvated nucleophile is much stronger than a solvated one [21]. Desolvation of nucleophilic species in the catalytic site of enzymes is considered as one more factor adding up to account for the efficiency of these systems [22].

Nevertheless, the catalytic site of an enzyme is far more complex than this and other contributions are clearly relevant to produce the, still unmatched, rate acceleration of a nuclease. In order to uncover other possible relevant contributions that could improve the catalytic efficiency of AuNPs-based nanozymes we have studied several new systems where we have varied the structure of the thiols used for the passivation of the nanoparticles and have also added small peptides flanking the metal center to further mimic the catalytic site of an enzyme. Both peptides used feature a guanidinium of arginine and one of them two serines. These amino acids play key roles in the catalytic sites of phosphate-cleaving enzymes [1]. Here we report our results.

## 2. Results and Discussion

### 2.1. Synthesis of the Thiols and Passivation of AuNPs 

**Figure 1:**
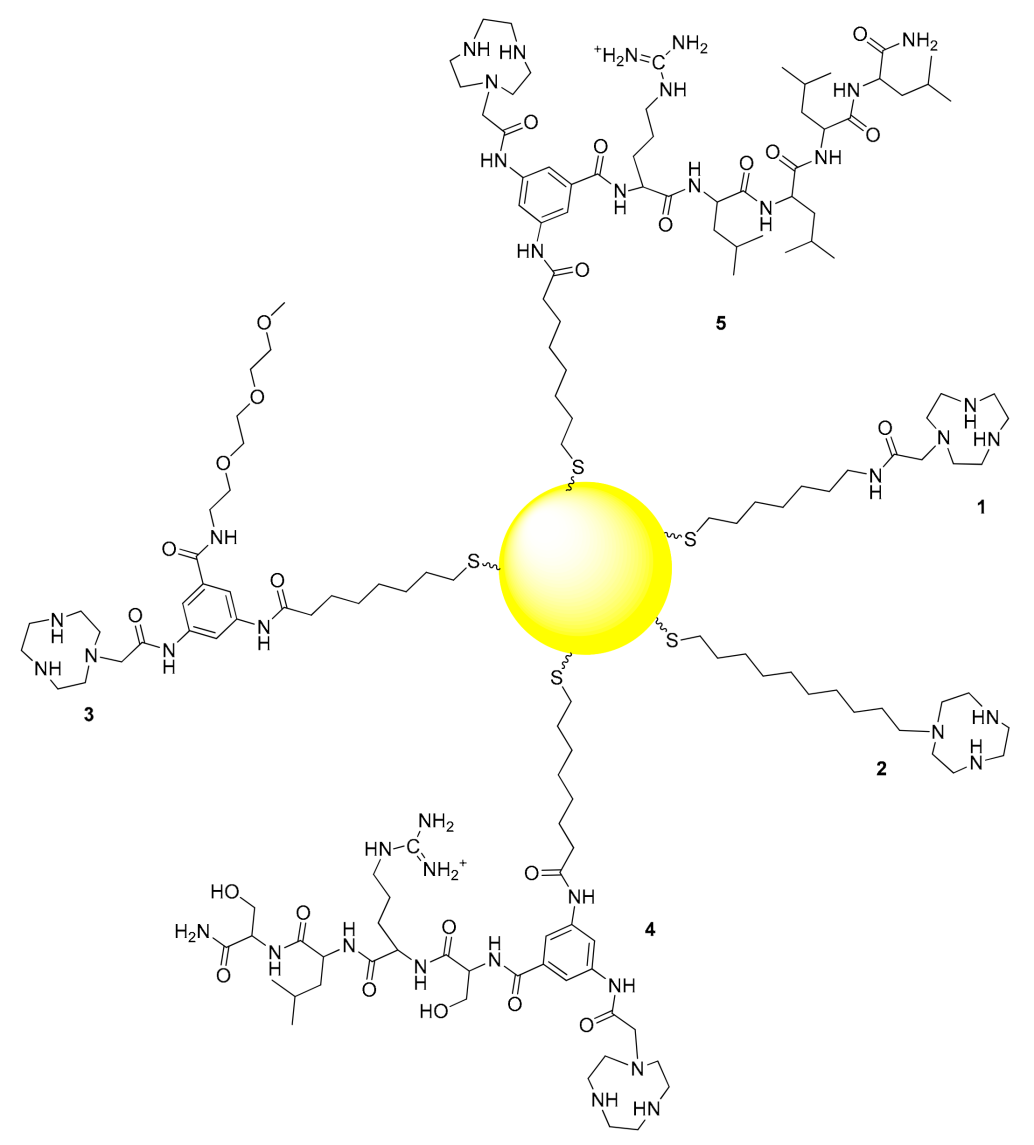
Thiols used for the passivation of the gold nanoparticles studied in this work.

We prepared five different AuNPs, each functionalized with one of the five different thiols reported in Figure 1. All are characterized by the presence of a TACN unit for complexation with Zn(II). In thiol 1 a C_7_ hydrocarbon chain was connected to the TACN unit via an amide bond. In thiol 2 the TACN was directly connected to the sulfhydryl group via a C_10_ hydrocarbon chain leading to the most hydrophobic monolayer. The remaining three thiols were Y-shaped with a 3,5-diamino benzoic acid derivative acting as the junction between three different arms: A thiolated unit for the attachment to the AuNP surface, a TACN ligand and a flanking polyether (3) or peptide (4 and 5). For the introduction of the units that could potentially assist the TACN-Zn(II) complex in the catalytic process we chose the Y-shaped thiol approach instead of functionalizing the AuNPs with two different thiols, to avoid their sorting on the passivating monolayer. The clustering of different thiols on the monolayer is a well known phenomenon that can lead to the formation of patches of the two different thiols on the same AuNPs or, in the extreme case, to the confinement of each of them in two different AuNPs [23,24,25,26,27,28]. The segregation of the thiols on the monolayer would result in the partial (or total) loss of the cooperativity between the different functional groups present on each of them. Such segregation was obviously not possible in the case of Y-shaped thiols 3–5.

**Scheme 1:**
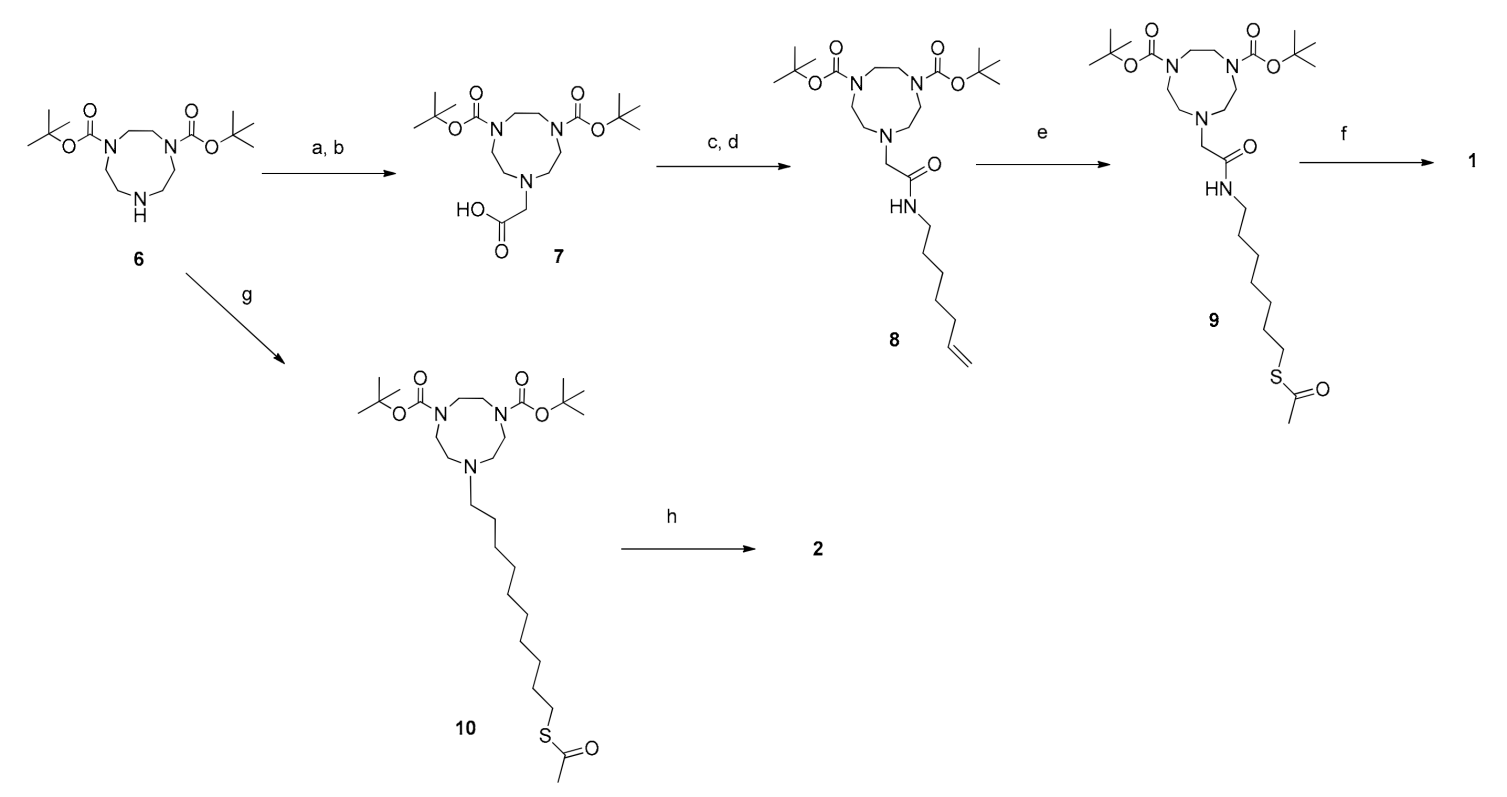
Synthetic route to thiols 1 and 2. a: 2-Br-methylacetate, Cs_2_CO_3_ in ACN(dry), N_2_, overnight, room temperature (rt); b: 1 M NaOH in MeOH, 2 h, rt; c: pentafluorophenol, EDC^.^HCl in DCM(dry), N_2_, overnight, rt; d: hept-6-en-1-ammonium chloride, DIPEA in DCM (dry), N_2_, overnight, rt; e: 2,2-dimethoxy-1,2-diphenylethan-1-one, thioacetic acid in MeOH, light irradiation (hv) = 254 nm, 1 h, rt; f: 6 M HCl in EtOH, 2 h, reflux; g: 10-Bromothioacetate, K_2_CO_3_, NaHCO_3_ in ACN, 4 h, 40 °C; h: 1 M HCl in EtOH, 2 h, reflux.

The synthesis of thiols 1 and 2 started from Boc-diprotected TACN and is reported in Scheme 1. In the case of 1 it was then alkylated with 2-bromomethylacetate. Formation of the amide with 1-amino-6-heptene followed by the thioene reaction [29] led, after deprotection, to the final product. Thiol 2 was obtained by alkylation of Boc-diprotected TACN with 10-bromodecyltioacetate followed by the hydrolysis of the thioacetate and deprotection. The syntheses of derivatives 3–5 were more complex and started from the selective protection of 3,5-diamino benzoic acid as the junction unit. This was followed by the stepwise functionalization with the three different arms. First, one amino group was reacted with octan-7-enoic acid. Subsequently, the diprotected triazacyclononane was connected to the second amino group via an amide bond. 

**Scheme 2:**
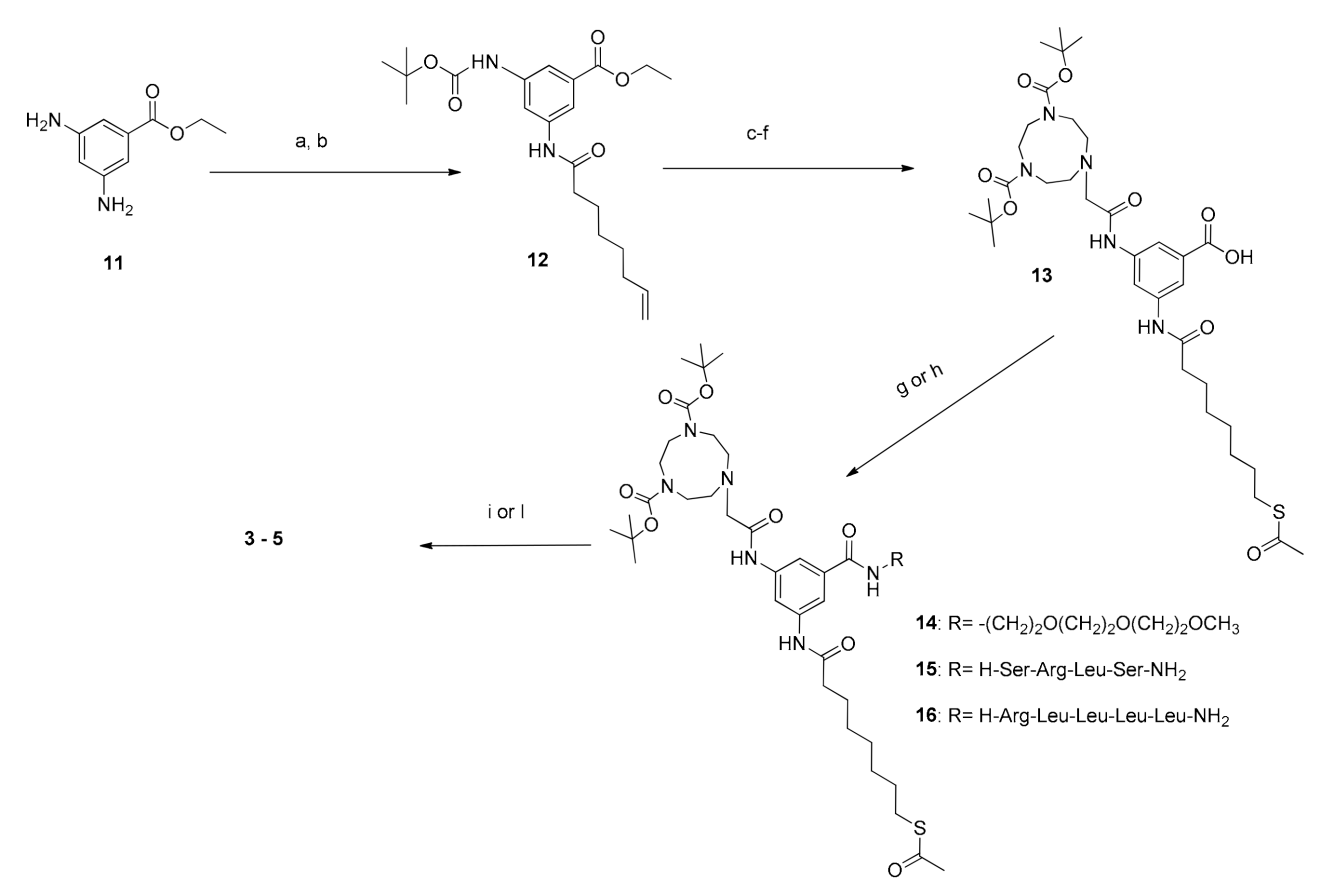
Synthetic route to Y-shaped thiols 3–5. a: Boc anhydride, Et_3_N, in dioxane/water, overnight, rt; b: octan-7-enoic acid, EDC^.^HCl, HOBt, Et_3_N, in ACN(dry), N_2_, overnight, rt; c: TFA in DCM, 2 h, rt; d: **7** is converted to the activated ester with pentafluorophenol, EDC^.^HCl in DCM(dry), N_2_, overnight, rt and then coupled in the presence of DIPEA, DCM(dry), N_2_, 2 days, reflux; e: 1 M NaOH in EtOH, 2 h, rt; f: Thioacetic acid, AIBN, in toluene, overnight, reflux; g: 2-(2-(2-methoxyethoxy)ethoxy)ethan-1-amine, Et_3_N, EDC^.^HCl, HOBt in DCM(dry), N_2_, overnight, rt, for the synthesis of 14; h: peptide, DIC, oxyma, 2,4,6-trimethylpyridine in DMF, overnight, RT, for 15 and 16; i: 95% TFA, 2.5% TRIS, 2.5% H_2_O, 2 h, rt, then CH_3_ONa in MeOH(dry), N_2_, 2 h, rt, for the synthesis of 3; l: 95% TFA, 2.5% TRIS, 2.5% H_2_O, 2 h, rt, then NH_3_ in MeOH(dry), N_2_, overnight, rt for the synthesis of 4 and 5.

Eventually the carboxylate group was converted into an amide by reaction with the proper compound: 1-amino tri(oxyethethylene)oxymethyl derivative for the synthesis of 3 and the *N*-terminus of the peptides for 4 and 5. The two peptides were obtained by conventional solid phase synthesis. Thioene reaction followed by full deprotection resulted in the obtainment of thiols 3–5. The strategy is outlined in Scheme 2. Key synthetic steps are reported in the Materials and Methods section while the full synthetic details are reported in the Supplementary Materials together with the original spectra of the products and selected intermediates.

To avoid oxidation, thiols 1–5 were deprotected immediately before their use for the passivation of the gold nanoparticles. The ca. 2 nm (gold core diameter) AuNPs were obtained following a procedure we have reported previously, which used a two-phase protocol with dioctylamine as a temporary AuNPs passivating agent [30]. AuNP1-5 (Figure 1) was obtained with good purity and was characterized by ^1^H-NMR, thermogravimetric analysis (TGA), UV-Vis spectroscopy and transmission electron microscopy (TEM). Characterization results are reported in the Supplementary Materials.

### 2.2. Kinetics of the Cleavage of HPNP in the Presence of the Zn(II) Complexes of AuNP1-5.

#### 2.2.1. Cooperativity between Functional Groups 

**Scheme 3:**
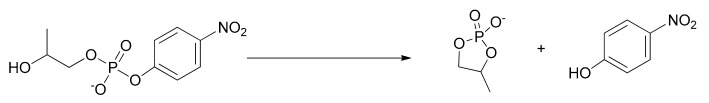
The cleavage of HPNP studied in this work.

2-Hydroxypropropyl *p*-nitrophenyphosphate, HPNP, was chosen as the substrate as it is a fair model for the cleavage of RNA. It allows the easy monitoring of the progress of its cleavage by following spectrophotometrically the *p*-nitrophenol (or *p*-nitrophenoxide, depending on the pH) formed during the reaction. The cyclic phosphate, 4-methyl-1,3,2-dioxaphospholan-2-olate-2-oxide, was the other product of the reaction, resulting from the intramolecular attack of the alcoholic –OH of HPNP to the phosphorus atom (Scheme 3). The process was analogous to that occurring in the cleavage of RNA with two important differences: a) The cyclic structure of the ribose present in RNA favors the intramolecular attack of the hydroxyl to the phosphorus of the phosphate ester; 2) *p*-nitrophenol (*p*-nitrophenoxide) is such a good leaving group that any information on the assistance in its departure by the catalyst is lost because this can hardly be the rate determining step of the reaction.

**Figure 2:**
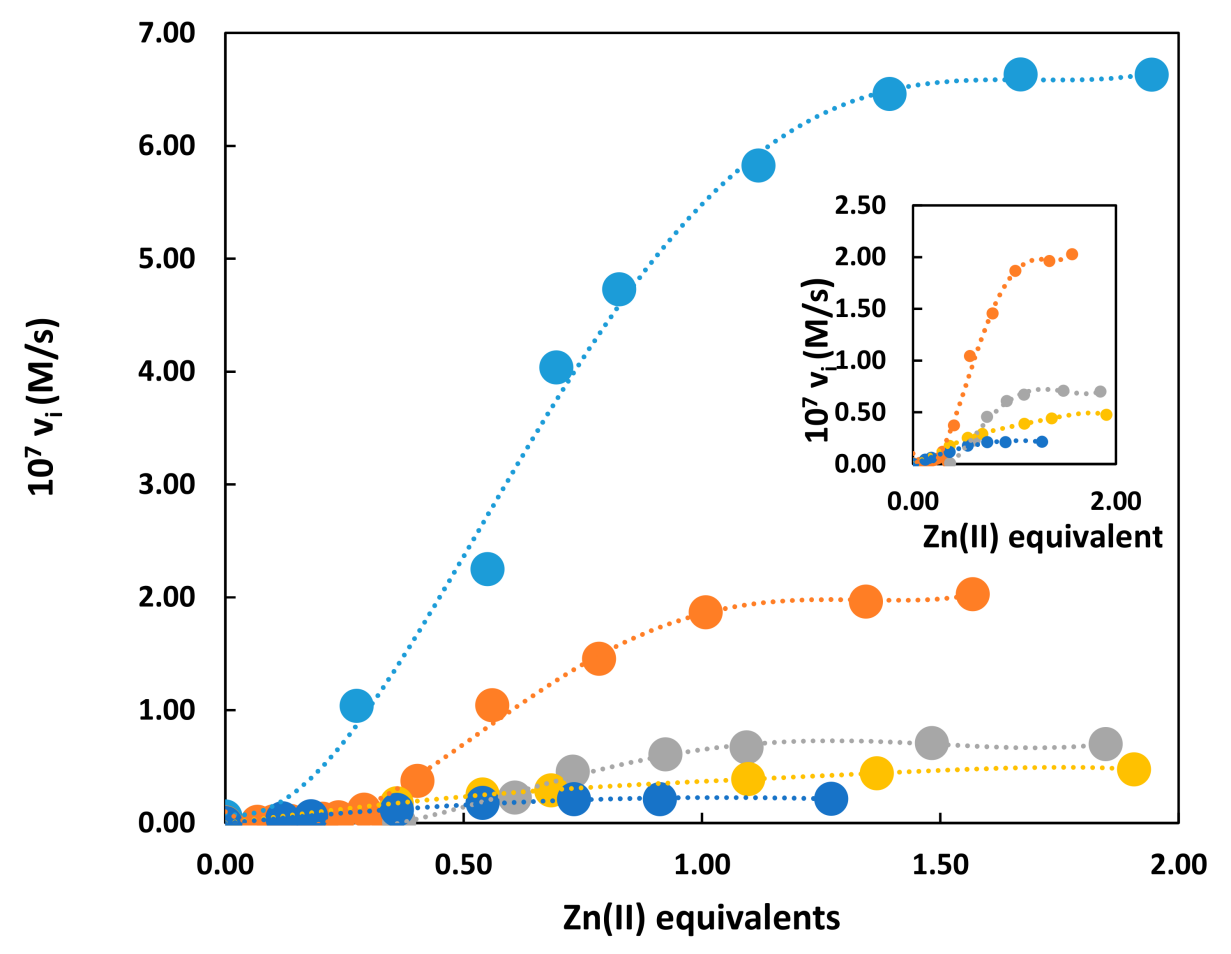
Titration kinetics obtained by adding Zn(NO_3_)_2_ to the solutions of the different gold nanoparticles (AuNPs). Conditions: pH = 7.5, 25 °C, [HPNP] = 1.0 × 10^−5^ M. Symbols code: AuNP1: Light blue; AuNP2: Orange; AuNP3: Gray; AuNP4: Yellow; AuNP5: Dark blue. Inset: Blown-up plot without the AuNP1 trace.

Titration kinetics in the presence of the different AuNP catalysts obtained by progressively adding Zn(II) to the nanoparticle solution show (Figure 2) that, in all cases, maximum catalytic activity is obtained when all triazacyclonane units present on the monolayer form the complex with the metal ion. The shape of kinetic titration curves for AuNP1-3 is sigmoidal while that of AuNP4,5 present a typical saturation profile (Figure 2, inset).

From previous studies we knew that for multivalent, Zn(II)-based catalysts as those constituted by nanoparticles or dendrimers, optimum catalysis is obtained through the cooperation of two Zn(II) ions [31]. The sigmoidal shape of the kinetic titration curves shown by AuNP1-3 fully supported such cooperativity. At low Zn(II) loading, the metal complexes were unable to form such dinuclear catalytic sites contrary to what happened under saturation conditions when all complexes were formed. The curves of Figure 2 were all normalized at the same concentration of Zn(II) ions. However, it should be noted that, in the case of the Y-shaped thiols, each Zn(II) complex was close to another functional group present in the other constituent moiety (peptide or tri(oxyethylene)oxymethyl derivative). This will make the cooperation between two of them in the catalytic process less likely to be achieved. Nevertheless, AuNP3 still presented a sigmoidal profile, an indication that, in spite of this arrangement in the monolayer, the major source of catalysis derived from the cooperativity between two Zn(II) ions (i.e., the triethyleneoxymethyl group was an uninvolved spectator). On the contrary, with the Y-shaped catalysts bearing the peptides it appeared they no longer relied on the cooperation between two metal centers. Both peptides of thiols 4 and 5 present an arginine in their sequence and hence a cationic guanidinium group: This group might be involved in the catalytic process. The guanidinium ion was catalytically non active in the absence of the Zn(II) ion but, with the progressive addition of the metal ion the catalytic activity increased till all triazacyclonane units formed the complex. We knew from our previous studies that a single metal ion is catalytically very little active [8]. It is thus reasonable to hypothesize that with AuNP4,5 the catalytic site comprised of a single metal ion and a flanking guanidinium group. A similar catalytic site arrangement has been reported by Casnati and Savio using calix[4]arenes [32,33].

#### 2.2.2. Michaelis–Menten Kinetics

**Figure 3:**
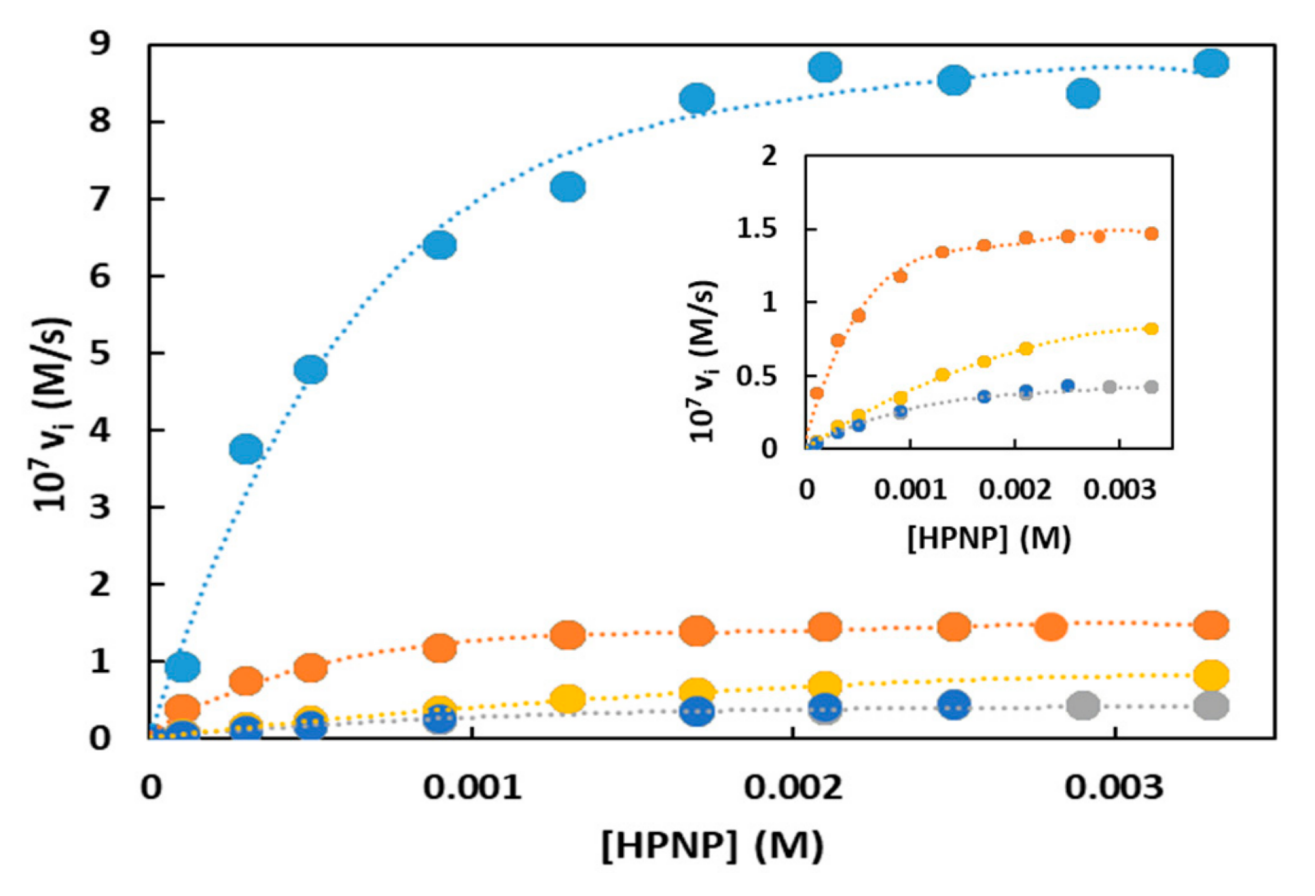
Michaelis–Menten kinetics obtained for the cleavage of HPNP by catalysts AuNP1-5. Conditions: [AuNP], (in triazacyclononane units) = [Zn(II)] = 2.5 × 10^−5^ M, [EPPS, buffer] = 1.0 × 10^−2^ M pH = 7.5, 25 °C. Symbols code: AuNP1: Light blue; AuNP2: Orange; AuNP3: Gray; AuNP4: Yellow; AuNP5: Dark blue. Inset: Blown-up plot without the AuNP1 trace.

The catalytic efficiency of the five nanoparticles studied were determined by performing HPNP cleavage kinetics under saturation conditions using a stoichiometric amount of Zn(II) with respect to the triazacyclononane units present on each nanoparticle. The results are reported in Figure 3 and Table 1. 

On the bases of the information acquired on the composition of the catalytic site in the different AuNPs (see previous Section 2.2.1), the concentration of catalyst was normalized for the species involved: Two Zn(II) ions for AuNP1-3 (bimetallic catalysis) and only one for AuNP4,5 (monometallic catalysis). The analysis of the data revealed that the substrate bound more strongly to AuNP1-2 than those passivated by the Y-shaped thiol. The dilution of the metal complexes on the monolayer was likely responsible for the lower affinity of HPNP for AuNP3 (compare the K_M_ for AuNP1-2 with that AuNP3). It is known that phosphates bind more strongly to cationic Zn(II) complexes than to ammonium ions [31,34]. Thus, the substitution of a Zn(II) complex with a guanidinium results in a lower affinity for the substrate (compare the K_M_ for AuNP1,2 with those of AuNP4,5). The k_cat_ values indicated that the best catalyst was AuNP1, which was 6.2-fold better than AuNP2. The comparison between AuNP1 and AuNP2 indicated that the amide functional group present in thiol 1 played a relevant role in the optimization of the catalytic site and that a hydrophobic hydrocarbon chain did not provide the only significant kinetic advantage. AuNP1 was more efficient than structurally similar AuNPs we had reported previously, representing one of the most effective catalysts for the cleavage of HPNP in water ever reported. The comparison between the dinuclear catalyst AuNP2 and the mononuclear one (AuNP4) indicated that a guanidinium ion could replace a metal ion but with lower efficiency (13.7- and 2.2-fold worse with respect to AuNP1 and AuNP2, respectively). It is difficult to make a proper comparison because on one side we preserved the amide bond connecting the triazacyclonanane to the rest of the molecule (as in AuNP1) while on the other we missed part of the hydrophobic contribution to catalysis. It is evident that several factors contribute to the improvement of the catalytic site and the comparison between monolayers with different functional groups is not straightforward. Two aspects emerge from the analysis of the kinetic data with the AuNPs functionalized with the two peptides (AuNP4,5). First it appeared that even a small change of the position of Arg (and hence of the guanidinium group) in the sequence was relevant; second that Ser did not appear to be involved. The last aspect was hardly surprising since the substrate had the possibility to take advantage of the intramolecular attack of the built-in hydroxyl (like in RNA). 

Second order rate constants (k_2_) take into consideration both k_cat_ and K_M_ and represent an evaluation of the catalysts both from the point of view of the substrate recognition and the performance of the catalytic site. For this reason, a bimetallic catalytic site, with a stronger affinity for the substrate, represents an advantage and the catalysts with the best second order rate constants were AuNP1,2, with AuNP1 ca. 4-fold better than AuNP2. Among the nanoparticles with the Y-shaped thiols, the better second order rate constant of AuNP3 (bimetallic catalytic site) than that of AuNP4 (monometallic catalytic site) indicated that the higher affinity of the dimetallic catalyst more than compensated for the slightly lower kinetic efficiency (expressed as k_cat_).

### 2.3. The Source of Catalysis in Zn(II)-Triazacyclononane-Functionalized Nanozymes

From the results reported above it is evident that the our nanozymes catalyzed the cleavage of HPNP following two different mechanisms, which were controlled by the different functional groups present in the thiols passivating the nanoparticles gold core. With AuNP1-3 the catalytic site comprised of two Zn(II) ions while with AuNP4,5 the catalytic site comprised of a single Zn(II) ion and a guanidinium ion of the arginine present in the peptide sequence of each of them [32,33]. Both mechanisms required the preliminary binding of the substrate to the nanozyme as proven by the Michaelis–Menten behavior of their kinetics. 

**Figure 4:**
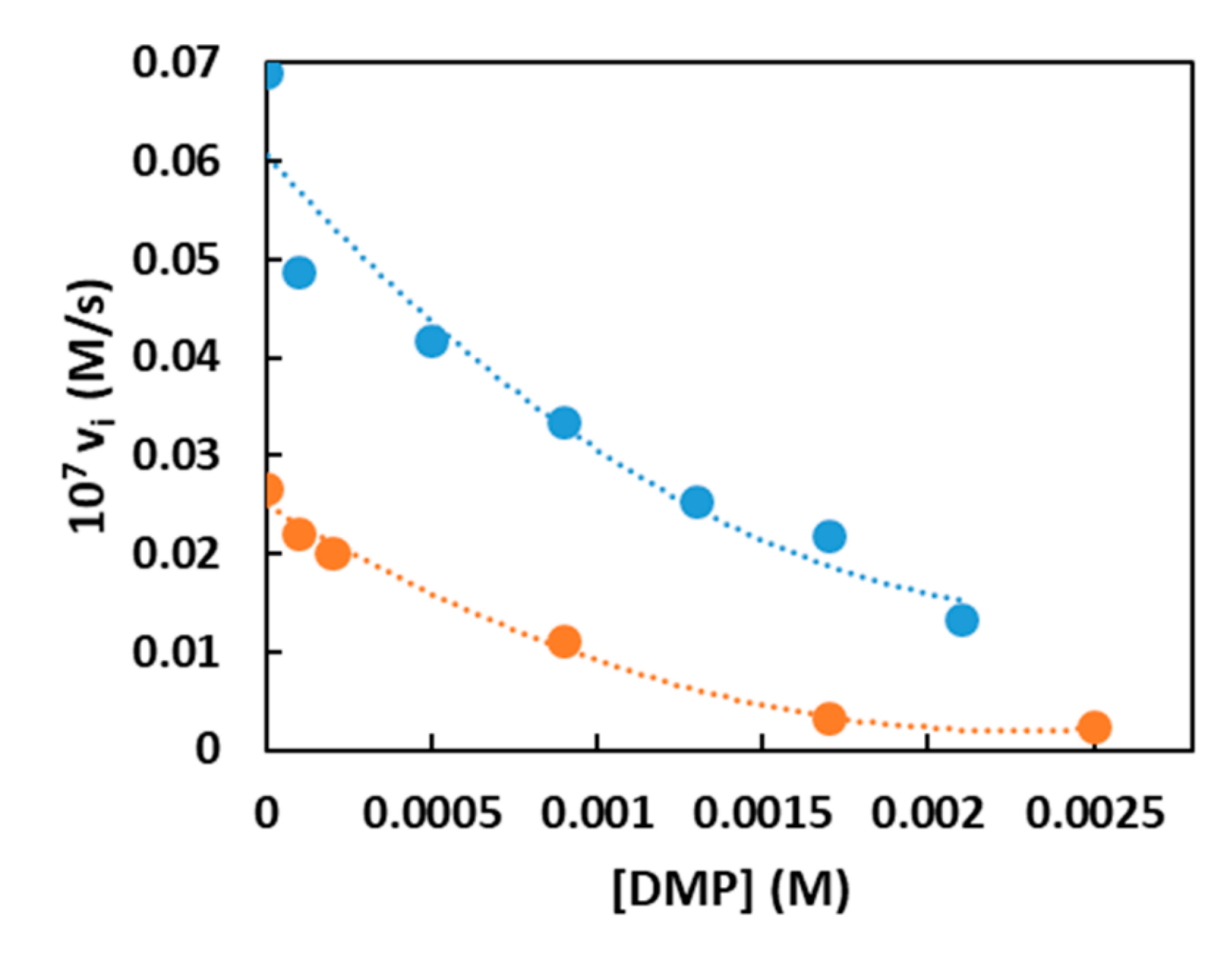
Inhibition kinetics obtained for catalysts AuNP1 and AuNP2 by progressive addition of dimethylphosphate (DMP). Conditions: [AuNP], (in thiol units) = [Zn(II)] = 2.5 × 10^−5^ M, [EPPS, buffer] = 1.0 × 10^−2^ M, [HPNP] = 1.0 × 10^−5^ M; pH = 7.5, 40 °C in H_2_O. Symbols code: AuNP1: Blue; AuNP2: Orange.

For AuNP1 and AuNP2 this was confirmed by inhibition experiments (Figure 4) obtained by adding increasing amounts of non-reactive dimethylphosphate (DMP) to the reaction solution. The apparent inhibition constant K_i_ was calculated to be 23.2 mM for AuNP1 and 38.5 mM for AuNP2. The fact that HPNP bound better to AuNP2 than to AuNP1 while the opposite was true for DMP, indicated that there was a significant hydrophobic contribution to binding with HPNP but that this was not transferred into catalytic efficiency. 

#### 2.3.1. Dinuclear Catalysts AuNP1-3

**Figure 5:**
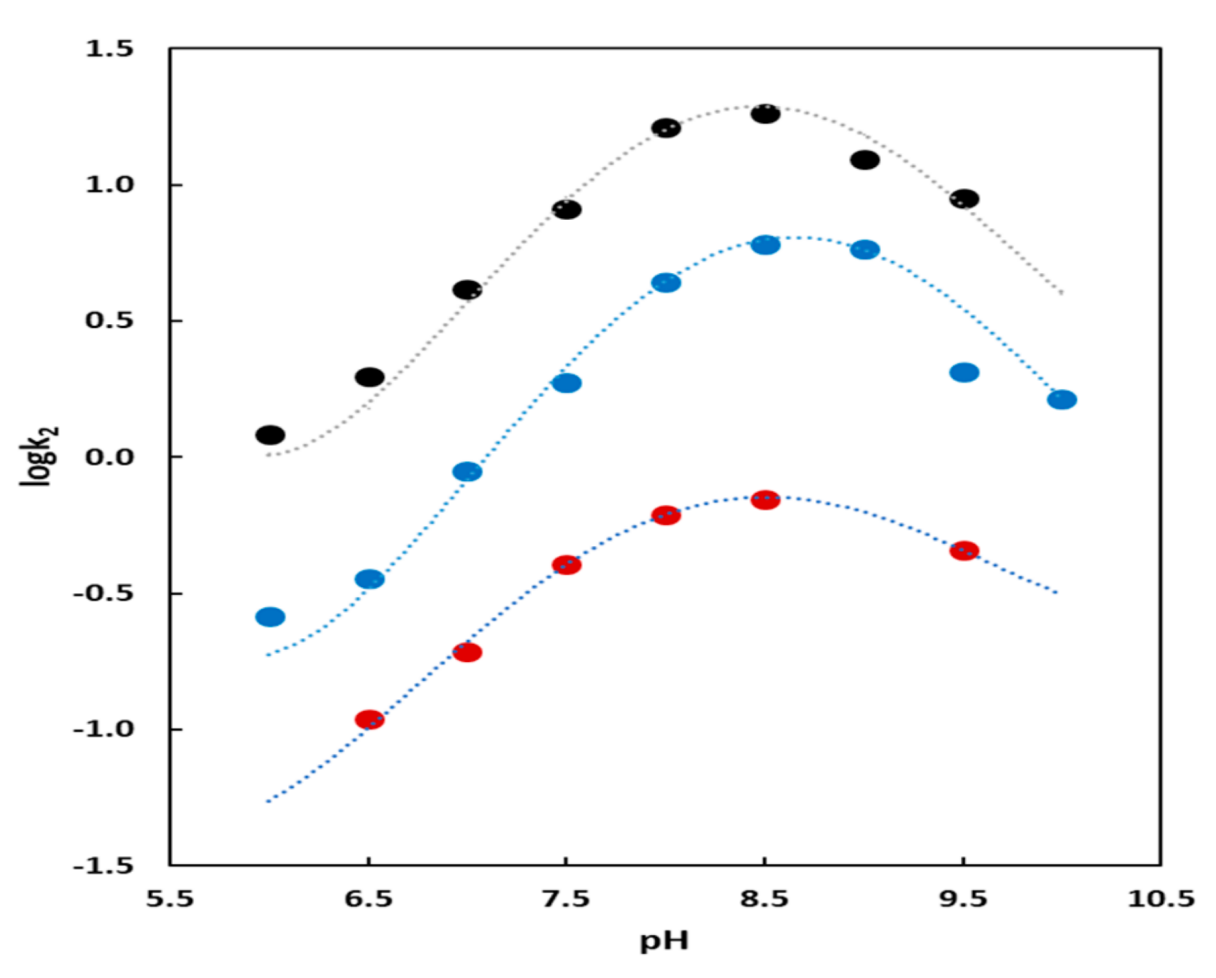
Dependence of the second order rate constant (k_2_) from pH for the catalysts AuNP-1, AuNP2 and AuNP4. The dotted lines represent the best fitting of the experimental data by using Equation (1). Black circles: AuNP2; blue circles: AuNP1 and red circles AuNP4. Conditions: [AuNP1 in thiol units] = [Zn(II)] = 1.7 × 10^−5^ M, [HPNP] = 1.0 × 10^−3^ M, 25 °C in H_2_O; [AuNP2]. (in thiol units) = [Zn(II)] = 3.47 × 10^−5^ M, [HPNP] = 7.0 × 10^−4^ M, 40 °C in H_2_O; [AuNP4] (in thiol units) = [Zn(II)] = 1.43 × 10^−5^ M, [HPNP] = 5.0 × 10^-4^ M, 40 °C in H_2_O; [buffers] = 1.0 × 10^−2^ M in all cases.

The kinetic profiles for catalysts AuNP1 and AuNP2 showed a bell-shaped dependence from pH (Figure 5). This graph is associated [35] with two deprotonation processes that have pKa 7.9 and 9.3 for AuNP1 and 7.8 and 9.2 for AuNP2 by fitting the data with Equation (1):

*k_2_* = *k*[C_o_]_tot_/((1 + [H^+^]/*K_a1_* + *K_a2_*/[H^+^]) + *k_H_*[H^+^]), (1)

where *K_a1_* and *K_a2_* are the first and second kinetically relevant deprotonation constant and *k_H_* is the acid catalyzed background reaction. *K_a1_*, beneficial to the catalytic process is associated with the deprotonation of a nucleophilic species, very likely the alcohol of HPNP. *K_a2_*, which is detrimental, is associated with the deprotonation of a Zn(II)-bound water molecule. The resulting Zn(II)-bound OH^-^ is less favorably displaced by the substrate than Zn(II)-bound H_2_O with consequent decreased formation of the required substrate–catalyst complex [36]. Kinetics performed in D_2_O (see Supporting Material) showed a dependence from pD superimposable to that from pH (H_2_O). This implies there was not a proton transfer (or H-bond formation) in the rate determining step of the reaction [6]. These kinetic studies excluded also any similar involvement by the amide bond connecting the TACN-Zn(II) complex to the thiolated hydrocarbon moiety. All experimental data so far discussed support a mechanism for these catalysts in which HPNP binds to a Zn(II) ion while a second metal ion deprotonates (not necessarily by direct coordination) the alcohol that acts as the intramolecular nucleophile to displace the *p*-nitrophenoxide leaving group [37]. Alternatively, the phosphate could bind to two metals and one of the two facilitated nucleophile deprotonation, as well. In any case, the proper proximity between the two cooperating metal complexes is a critical aspect of this mechanism. Thus, it is not surprising that AuNP3 is a worse catalyst than AuNP1 because of the “dilution” in the monolayer passivating the nanoparticles of the Zn(II)-TACN complex with the triethyleneoxymethyl units. The puzzling question is why AuNP2 is also a poorer catalyst than AuNP1. What we know from previous studies from our group [38] is that the amide group present in 1 was involved in strong H-bonding within the monolayer. This will likely result in a better packing of the molecules with consequent more precise control on the relative position of the Zn(II)-TACN complexes leading to enhanced cooperativity between them. This is an appealing, although speculative, explanation that deserves further investigation.

#### 2.3.2. Mononuclear Catalysts AuNP4 and AuNP5

The rate vs. pH profile for the nanoparticles passivated with the Y-shaped-thiol 4 with the peptides flanking the Zn(II)-TACN (Figure 5) also showed a bell-shaped profile. By using Equation (1) the two kinetically relevant deprotonation constant had a pKa of 7.5 and 10.1. The first one was again associated with the activation of the nucleophile, the alcoholic function of the substrate. The value was slightly lower than those obtained in the analogous plots for AuNP1 and AuNP2 likely as the consequence of the increase of the local pH due to the presence of the guanidinium cation in analogy to what happens with cationic micelles or vesicles and cationic nanoparticles, as well. A local increase of the pH would result in an apparent decrease of the pKa of the nucleophile, as we observed. On the contrary the value of the second pKa was higher than those obtained for AuNP1 and AuNP2. We associated it to the deprotonation of the guanidinium ion for which the reported pKa was 12.5 and would decrease to 10.1 because of the presence of the Zn(II) complexes in the monolayer (possibly through an indirect, weak coordination). The deprotonation of the guanidinium group to guanidine results in the loss of the electrostatic interaction for the binding of the phosphate of HPNP. The free base could also bind to the single metal ion. Accordingly, with AuNP4 the mechanism was different from the previous ones because HPNP was brought in close proximity to the metal complex, where the activation of the nucleophile occurred, through the interaction with the guanidinium cation and not with a second metal ion. The role of guanidinium would be, accordingly, dual: Decreasing the local pH and assisting in the binding of the phosphate to the catalyst [39]. A possible role as a general-acid catalyst cannot be, however, ruled out [40].

## 3. Materials and Methods 

### 3.1. General

Solvents were purified by standard methods. All commercially available reagents and substrates were used as received. Thin Layer Chromatography (TLC) analyses were performed using Merck 60 F254 precoated silica gel glass plates. Column chromatography was carried out on Macherey–Nagel silica gel 60 (70–230 mesh). NMR spectra were recorded using a Bruker AC250F spectrometer operating at 250 MHz for ^1^H and 62.9 MHz for ^13^C and a Bruker AV300 operating at 300 MHz for ^1^H (Bruker Corporation, Billerica, MA, USA). Chemical shifts were reported relative to internal Me_4_Si. ESI-MS mass spectra were obtained with an Agilent Technologies LC/MSD Trap SL mass spectrometer. UV-Visible spectra and kinetic traces were recorded on Perkin Elmer Lambda 16 and Lambda 45 spectrophotometers (PerkinElmer Inc., Waltham, MA, USA) equipped with thermostated multiple cell holders. Kinetics were followed by monitoring the change of absorbance of formed *p*-nitrophenol at 317 nm (pH < 6.2) or *p*-nitrophenolate at 400 nm (pH > 6.2). They were started by adding 20 µL of substrate in CH_3_CN to the cuvette containing 2 mL of buffered solution with the proper amount of catalyst. The buffer components were used as supplied by the manufacturers: Acetic acid (Sigma-Aldrich, Saint Luis, MO, USA), 2-morpholinoethanesulfonic acid (MES, Fluka Thermo Fisher Scientific Inc., Waltham, MA, USA), 4-(2-hydroxyethyl)-1-piperazineethanesulfonic acid (HEPES, Sigma-Aldrich, Saint Luis, MO, USA), 4-(2-hydroxyethyl)-1-piperazinepropanesulfonic acid (EPPES, Sigma-Aldrich, Saint Luis, MO, USA), 2-[*N*-cyclohexylamino]ethanesulfonic acid (CHES, Sigma-Aldrich, Saint Luis, MO, USA), 3-[cyclohexylamino]1-propanesulfonic acid (CAPS, Sigma-Aldrich, Saint Luis, MO, USA).

The detailed synthesis of thiols 1–5 used for the passivation of the AuNPs is reported in the Supplementary Materials along with their characterization and key spectroscopic details.

### 3.2. Preparation of the AuNPs

The procedure used followed a reported protocol [30]. Thus, an aqueous solution of HAuCl_4_·3H_2_O (1.0 eq.) was extracted with tetraoctylammonium bromide (3.0 eq.) previously dissolved in degassed toluene. To the red-orange organic phase, dioctylamine (20 eq.) was added. The mixture was vigorously stirred at room temperature under nitrogen for 30 min. During that time the color changed from an orange to colorless. The flask was placed in an ice-bath and then aqueous solution of NaBH_4_ (10 eq.) was rapidly added. The color changed immediately to brownish-black due to nanoparticles formation. After 2 hr of stirring, a small amount of left water was removed. Keeping the reaction under an ice-bath, desired thiol (2.0 eq.) dissolved in methanol was added to the solution and then the reaction was left stirring for additional 2 h. AuNPs were purified by their sonication and washing with different solvents (hexane, toluene, petroleum ether, ethyl acetate and methanol). Then, their concentrated solution was applied on the gel permeation chromatography with Sephadex G-25 resin.

## 4. Conclusions

Our work represents an important step in the understanding of the role of different functional groups and of the medium as well in affecting the catalytic activity of nanozymes in a phosphate ester transesterification. The results reaffirmed the critical role played by two metal ions in the catalytic site and the desolvation of the nucleophile. They also indicated that a decrease of freedom of the metal centers was useful in increasing their cooperation. We have also shown that a guandinium ion could replace a metal ion although this resulted in a slight decrease of catalytic efficiency of the nanozyme. Overall, the confinement of catalytic units in the monolayer passivating the gold cluster of a nanoparticle represented a significant advantage with respect to other catalysts featuring similar catalytic sites. Indeed our Zn(II) nanozymes remained among the best performing catalysts for the cleavage of the model substrate HPNP. AuNP1 showed a *k_2_* that was 1.3 fold better than the best performing nanozyme [20] and 6.2 fold better than the most efficient dinuclear Zn(II) complex reported so far [41].

## Figures and Tables

**Table 1:** Kinetic parameters obtained from the Michaelis–Menten analysis of the cleavage of HPNP by catalysts AuNP1-5.

Catalyst	k_cat,_ s^−1, a^	k_2,_ l × mol^−1^ × s^−1, b^	K_M,_ mM	k_rel_^c^
**AuNP1**	0.193	331	0.58	9.7 × 10^5^
**AuNP2**	0.031	81.6	0.38	1.5 × 10^5^
**AuNP3**	0.010	9.4	1.1	5.3 × 10^4^
**AuNP4**	0.014	5.1	2.7	6.9 × 10^4^
**AuNP5**	0.006	3.85	1.7	3.3 × 10^4^

^a^ Normalized for the theoretical Zn(II) concentration in the catalytic site ([Zn(II)]/2 for dinuclear AuNP1-3 and [Zn(II)] for mononuclear AuNP4-5); ^b^ k_2_ = k_cat_/K_M_; ^c^ k_rel_ = k_cat_/k_uncat_; k_uncat_ = 2.0 × 10^−7^ s^−1^.

## Supplementary Materials

The following are available online: detailed synthesis of thiols **1**–**5** and of the peptides by solid phase; Figures S1–S10 reporting the characterization of each AuNP via NMR, TGA, TEM; Figure S11 reporting the pH vs. pD kinetic data.
