# Supplementary material for: Factors Influencing the Activity of Nanozymes in the Cleavage of an RNA Model Substrate
Source: Molecules. 2019 Aug 1;24(15):2814. doi: 10.3390/molecules24152814 (PMC6696475; doi:10.3390/molecules24152814)
Supplement: Supplementary file 1 [file molecules-24-02814-s001.pdf]

Supplementary Material

for

Factors Influencing the Activity of Nanozymes in the Cleavage of an RNA Model Substrate

by

Joanna Czescik[1], Susanna Zamolo[2], Tamis Darbre[2,*], Fabrizio Mancin[1,*] and Paolo Scrimin[1,*]

1 University of Padova, Department of Chemical Sciences, via Marzolo, 1 35131 Padova, Italy
2 University of Bern, Department of Chemistry and Biochemistry, Freiestrasse 3, CH-3012 Bern, Switzerland

Content

1. **The synthesis of thiol (1)** **S2**
2. **The synthesis of thiol (2)** **S5**
3. **The synthesis of thiols (30, 4, 5)** **S6**
4. **The solid-phase synthesis of peptides** **S11**
5. **The synthesis of 2-hydroxypropyl *p*-nitrophenyl phosphate (34)** **S12**
6. **The synthesis of gold nanoparticles** **S13**
7. **Characterization of AuNP1** **S14**
8. **Characterization of AuNP2** **S16**
9. **Characterization of AuNP3** **S18**
10. **Characterization of AuNP4** **S20**
11. **Characterization of AuNP5** **S22**
12. **pH vs pD dependence of the rate of cleavage of HPNP** **S24**

1. The synthesis of thiol (1)

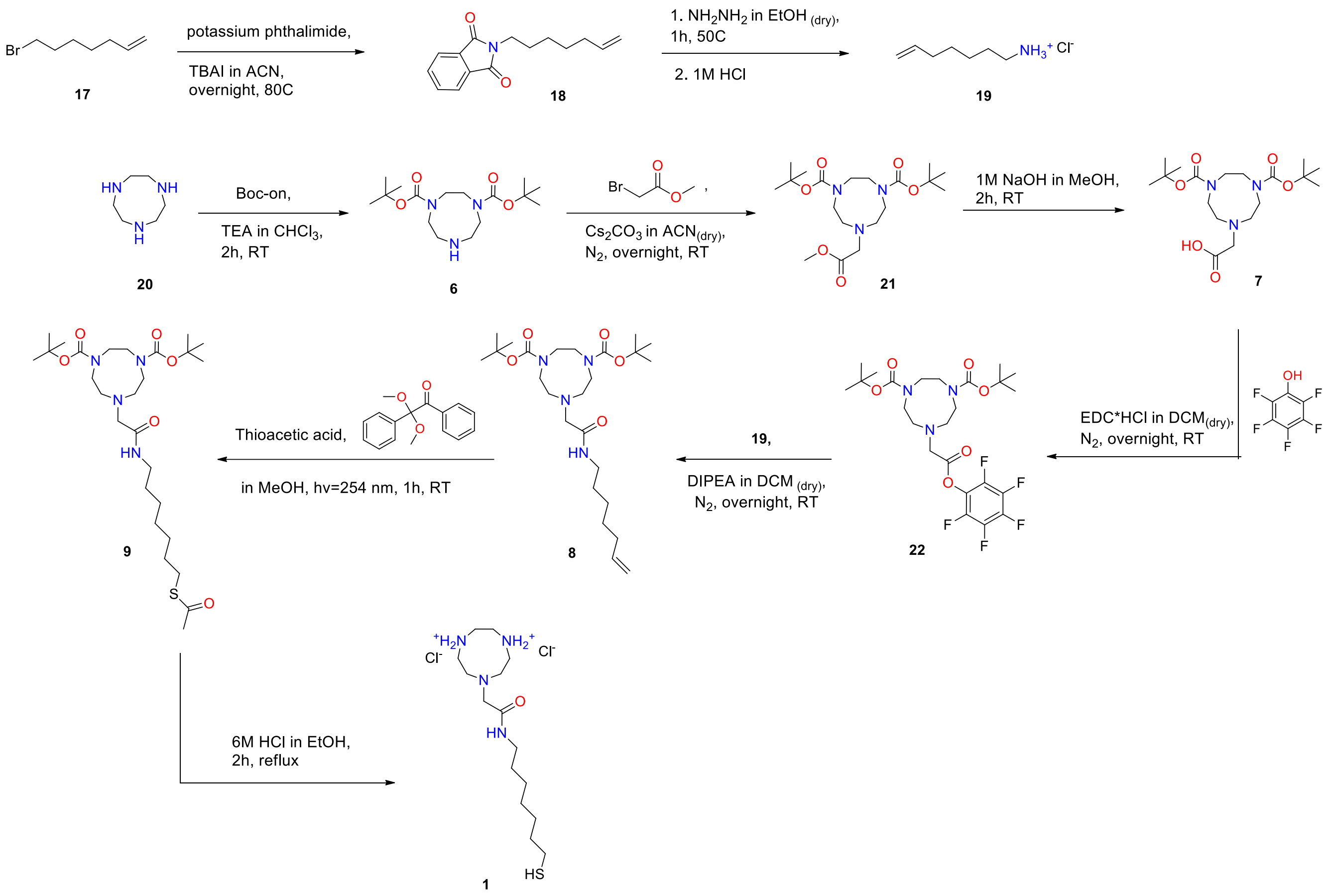

2-(Hept-6-en-1-yl)isoindoline-1,3-dione (18): In a round-bottom flask, tetrabutylammonium iodide (0.02 eq.) and potassium phthalimide (2.0 eq.) were suspended in acetonitrile. **17** (1.0 eq.) was added dropwise and the resulting mixture was heated up to 80˚C for exactly 24 hours. Once the mixture reached room temperature, it was diluted with diethyl ether and passed through a celite plug. The filtrate was concentrated in vacuo and the yellow residue was purified by column chromatography (hexane/ethyl acetate 95/5) giving the desired product as a white solid (83% yield).

^{1}H-NMR ($CDCl_3$, 500 MHz), δ: 7.85-7.82 (m, 2H), 7.72-7.69 (m, 2H), 5.82-5.76 (m, 1H), 5.00-4.91 (m, 2H), 3.68 (t, 2H), 2.04 (q, 2H), 1.68 (m, 2H), 1.45-1.34 (m, 4H)

^{13}C-NMR ($CDCl_3$, 500 MHz), δ: 168.9, 139.4, 134.2, 132.6, 123.6, 114.7, 36.44, 34.06, 29.12, 27.11

Hept-6-en-1-amonium chloride (19): In a glass tube **18** (1.0 eq.) was dissolved in dry ethanol and heated up to 50˚C. Hydrazine (NH_2NH_2; 1.5 eq.) was added and the resulting mixture was stirred at reflux for 1 hour under nitrogen. The obtained white slurry was quenched with 6M hydrochloric acid and stirred for an additional 30 minutes. The white solid was filtered off and washed several times with ethanol. The remaining filtrate was concentrated in vacuo and the residual aqueous solution was alkalized with 1M sodium hydroxide (pH = 9) and then extracted with chloroform. The collected

organic phases were then extracted with 1M hydrochloric acid, water phases concentrated in vacuo resulting with desired product as a pale-yellow solid (86% yield).

^{1}H-NMR (D_2O, 500 MHz), δ: 5.95-5.89 (m, 1H), 5.09-4.99 (m, 2H), 3.00 (t, 2H), 2.11-2.07 (m, 2H), 1.70-1.66 (m, 2H), 1.45-1.38 (m, 4H)
^{13}C-NMR (D_2O, 500 MHz), δ: 139.7, 114.3, 39.45, 32.70, 27.41, 26.48, 25.01

Di-tert-butyl-1,4,7-triazonane-1,4-dicarboxylate (6): In a round-bottom flask, the white powder of **20** (1,4,7- triazacyclononane; TACN; 1.0 eq.) together with triethylamine (TEA; 3.0 eq.) was dissolved in chloroform and cooled in an ice bath. [2-(tert-butoxycarbonyloxyimino)-2-phenylacetonitrile] (2.0 eq.) dissolved in chloroform was added dropwise over the course of 5 hours. After the addition was complete, the mixture was stirred at room temperature for additional 2 hours. Afterwards the solvent was evaporated and remaining yellow oily residue was washed with 5% aqueous solution of sodium carbonate, brine and 10% aqueous solution of citric acid. The acidic aqueous phase was alkalized with 1M sodium hydroxide to pH 11 and then extracted with chloroform. The chloroform was evaporated and the pure product collected as a clear oil (92% yield).

^{1}H-NMR ($CDCl_3$, 500 MHz), δ: 3.46-3.39 (m, 4H), 3.26-3.29 (m, 4H), 2.89 (m, 4H), 1.45 (s, 18H)
^{13}C-NMR ($CDCl_3$, 500 MHz), δ: 156.0, 155.8, 79.8, 79.7; 52.5, 52.4, 49.8, 49.5, 48.2, 48.09, 28.53
ESI-MS (m/z): 330.3 $[M + H]^+$

Di-tert-butyl-7-(2-methoxy-2-oxoethyl)-1,4,7-triazonane-1,4-dicarboxylate (21): In a two-neck round-bottom flask previously flushed with nitrogen, **6** (1.0 eq.) was dissolved in dry acetonitrile. Then sodium carbonate (3.0 eq.) and methyl bromoacetate (1.0 eq.) were added. The mixture was stirred overnight at room temperature under nitrogen. Acetonitrile was evaporated and the oily crude was purified by column chromatography (petroleum ether/ ethyl acetate 80/20-70/30) yielding the desired product as a colorless oil (83% yield).

^{1}H-NMR ($CDCl_3$, 500 MHz), δ: 3.75-3.73 (t, 3H), 3.54-3.51 (m, 7H), 3.30 (m, 4H), 2.89 (m, 4H), 1.55 (s, 18H)
^{13}C-NMR ($CDCl_3$, 500 MHz), δ: 172.8; 156.0, 79.78, 56.35, 54.56, 54.08, 53.85, 51.64, 51.09, 50.23, 49.80, 28.87
ESI-MS (m/z): 402.3 $[M + H]^+$

2-(4,7-Bis(tert-butoxycarbonyl)-1,4,7-triazonan-1-yl)acetic acid (7): In a round-bottom flask placed in an ice bath, **21** (4.7 mmol) was dissolved in methanol (40 ml). 1M sodium hydroxide (40 mL) was added dropwise. After the addition, the reaction was stirred for 1 hour at room temperature. Next, methanol was evaporated and the aqueous phase was acidified with 10% aqueous solution of potassium hydrogen sulfate until pH 3 was reached. The product was extracted with ethyl acetate and the collected organic phases were dried over sodium sulfate. After evaporation of the solvents, the desired product was isolated as a white solid (85% yield).

^{1}H-NMR ($CDCl_3$, 500 MHz), δ: 3.44 – 3.31 (m, 10H), 2.83-2.73 (m, 4H), 1.45 (s, 18H)
^{13}C-NMR ($CDCl_3$, 500 MHz), δ: 170.6, 155.3, 80.85, 60.52, 59.74, 53.99, 53.54, 49.87, 48.81, 46.83, 28.34
ESI-MS (m/z): 388.3 $[M + H]^+$

Di-tert-butyl-7-(2-oxo-2-(perfluorophenoxy)ethyl)-1,4,7-triazonane-1,4-dicarboxylate (22): In a two-neck round-bottom flask previously flushed with nitrogen, **7** (1.0 eq.) and pentafluorophenol (1.6 eq.) were dissolved in dry DCM and placed in an ice bath. When the temperature reached 0°C, N-(3-Dimethylaminopropyl)-N′-ethyl-carbodiimide hydrochloride (EDC*Cl; 1.6 eq.) was added. The mixture was stirred overnight at room temperature under nitrogen. The organic solution was washed with saturated aqueous solution of potassium hydrogen sulfate and brine and then dried over magnesium sulfate. After evaporation of the solvents, a brownish oil was purified by column chromatography (dichloromethane/methanol 98/2). The desired product was obtained as a white foam (86% yield).

^{1}H-NMR (CDCl$_3$, 500 MHz), δ: 3.77 (m, 2H), 3.42-3.37 (m, 4H), 3.25-3.21 (m, 4H), 2.84 (m, 4H), 1.45 (s, 18H)
^{13}C-NMR (CDCl$_3$, 500 MHz), δ: 167.7, 155.5, 141.8, 140.2, 139.8, 138.6, 138.2, 136.7, 79.62, 55.27, 53.71, 53.16, 51.37, 50.63, 50.04, 49.27, 28.20
ESI-MS (m/z): 554.3 [M + H]$^+$

Di-tert-butyl-7-(2-(hept-6-en-1-ylamino)-2-oxoethyl)-1,4,7-triazonane-1,4-dicarboxylate (8): In a two-neck round-bottom flask previously purged with nitrogen, **19** (1.0 eq.) and **22** (1.0 eq.) were dissolved in dry DCM. The flask was placed in an ice bath and after 15 minutes N,N-Diisopropylethylamine (DIPEA; 3.0 eq.) was added dropwise. After the whole addition, the mixture was stirred at reflux overnight. The mixture was concentrated in vacuo and purified by column chromatography (chloroform/ ethyl acetate 80/20-70/30). A clear oil was obtained (80% yield).

^{1}H-NMR (CD$_3$OD, 500 MHz), δ: 5.85-5.77 (m, 1H), 5.02-4.92 (m, 2H), 4.49 (s, 1H), 3.52-3.41 (m, 6H), 3.23-3.20 (m, 4H), 2.80-2.70 (m, 4H), 2.09-2.02 (m, 2H), 1.55-1.50 (m, 20H), 1.44-1.30 (m, 6H)
ESI-MS (m/z): 483.5 [M + H]$^+$

Di-tert-butyl-7-(2-((7-(acetylthio)heptyl)amino)-2-oxoethyl)-1,4,7-triazonane-1,4-dicarboxylate (9): In a 3 mL quartz cuvette, 2,2-dimethoxy-1,2-diphenylethan-1-one (0.05 eq.) was dissolved in 2 mL of degassed methanol. Then **8** (1.0 eq.) was added and the solution was treated with nitrogen flow for 15 minutes. Next, thioacetic acid (3.5 eq.) was added to the solution. The reaction was left stirring for 5 hours upon irradiation at 254 nm. Following this, the crude was concentrated, washed several times with methanol and purified by column chromatography. The elution was performed using dichloromethane/methanol 90/10. The desired product formed a yellow solid (80% yield).

^{1}H-NMR (CD$_3$OD, 500 MHz), δ: 3.52-3.40 (m, 6H), 3.22-3.20 (m, 4H), 2.86 (t, 2H), 2.70 (m, 4H), 2.30 (s, 3H), 1.56-1.40 (m, 23H), 1.34-1.27 (m, 7H)
ESI-MS (m/z): 559.3 [M + H]$^+$

7-(2-((7-Mercaptoheptyl)amino)-2-oxoethyl)-1,4,7-triazonane-1,4-diium chloride (1): In a round-bottom flask, **9** (0.1 mmol) was dissolved in a small amount of ethanol. Then an equal amount of 6M hydrochloric acid was added dropwise. After the complete addition, the resulting mixture was refluxed for 2 hours. The solvents were evaporated and the crude was dried under high vacuum.

Obtained product was used *in situ* without any additional purification in the synthesis of gold nanoparticles (**AuNP1**).

2. The synthesis of thiol (2)

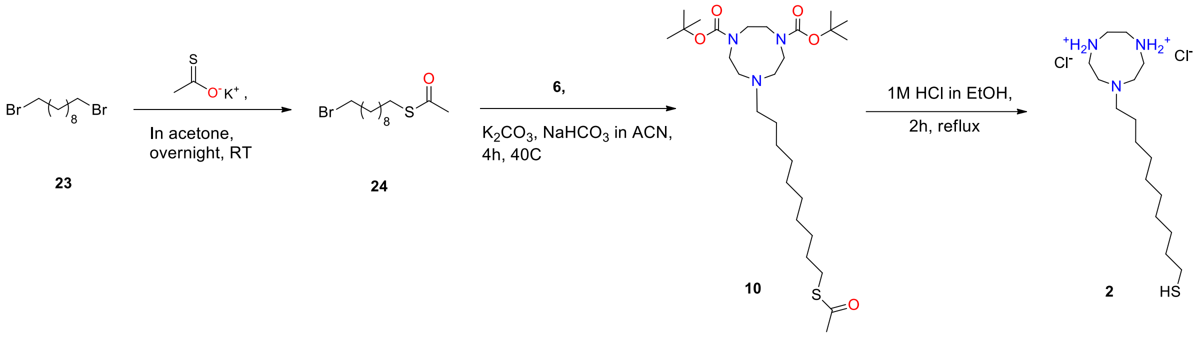

S-(10-Bromodecyl) ethanethioate (24): In a round-bottom flask, **23** (1.0 eq.) was dissolved in acetone. Potassium thioacetate (1.0 eq.) together with an additional portion of acetone was added. This resulting mixture was stirred overnight at room temperature. Afterwards the solution was concentrated, extracted with ethyl acetate and dried over a Na_2SO_4. After solvent evaporation, the remaining orange oil was purified by column chromatography (petroleum ether/ethyl acetate 98/2-96/4). The desired product was obtained as a white solid (46% yield).

^{1}H-NMR ($CDCl_3$, 500 MHz), δ: 3.40 (t, 2H), 2.86 (t, 2H), 2.32 (s, 3H), 1.83 (q, 2H), 1.56 (q, 2H), 1.56-1.28 (m, 12H)

^{13}C-NMR ($CDCl_3$, 500 MHz), δ: 196.51, 34.49, 33.19, 31.07, 29.87, 29.72, 29.52, 29.44, 29.16, 29.11, 28.53

Di-tert-butyl-7-(10-(acetylthio)decyl)-1,4,7-triazonane-1,4-dicarboxylate (10): In a round-bottom flask previously purged with nitrogen, potassium carbonate (3.0 eq.) and sodium bicarbonate (3.0 eq.) were suspended in dry MeCN. Then **24** (1.2 eq.) and **6** (1.0 eq.) were added. The mixture was stirred at 60˚C for 4 hours. Afterwards, the solvent was removed and resulting oily crude was purified by column chromatography (dichloromethane/methanol 97/3). The desired product was isolated as a yellow oil (40% yield).

^{1}H-NMR (CD_3OD, 500 MHz), δ: 5.85-5.78 (m, 1H), 5.01-4.94 (m, 2H), 3.48-3.21 (m, 8H), 2.63-2.46 (m, 4H), 2.06 (m, 2H), 1.85 (q, 2H), 1.48-1.27 (m, 30H)
ESI-MS (m/z): 544.3 $[M + H]^+$

7-(10-Mercaptodecyl)-1,4,7-triazonane-1,4-diium chloride (2): In a round-bottom flask **10** (0.1 mmol) was dissolved in a small amount of ethanol. An equal amount of 6M hydrochloric acid was added dropwise. After the addition, the resulting mixture was refluxed for 2 hours. Afterwards, the solvents were evaporated and the crude was dried under high vacuum. Obtained product was used *in situ* without any additional purification in the synthesis of gold nanoparticles (**AuNP2**).

3. The synthesis of thiols (30, 4, 5)

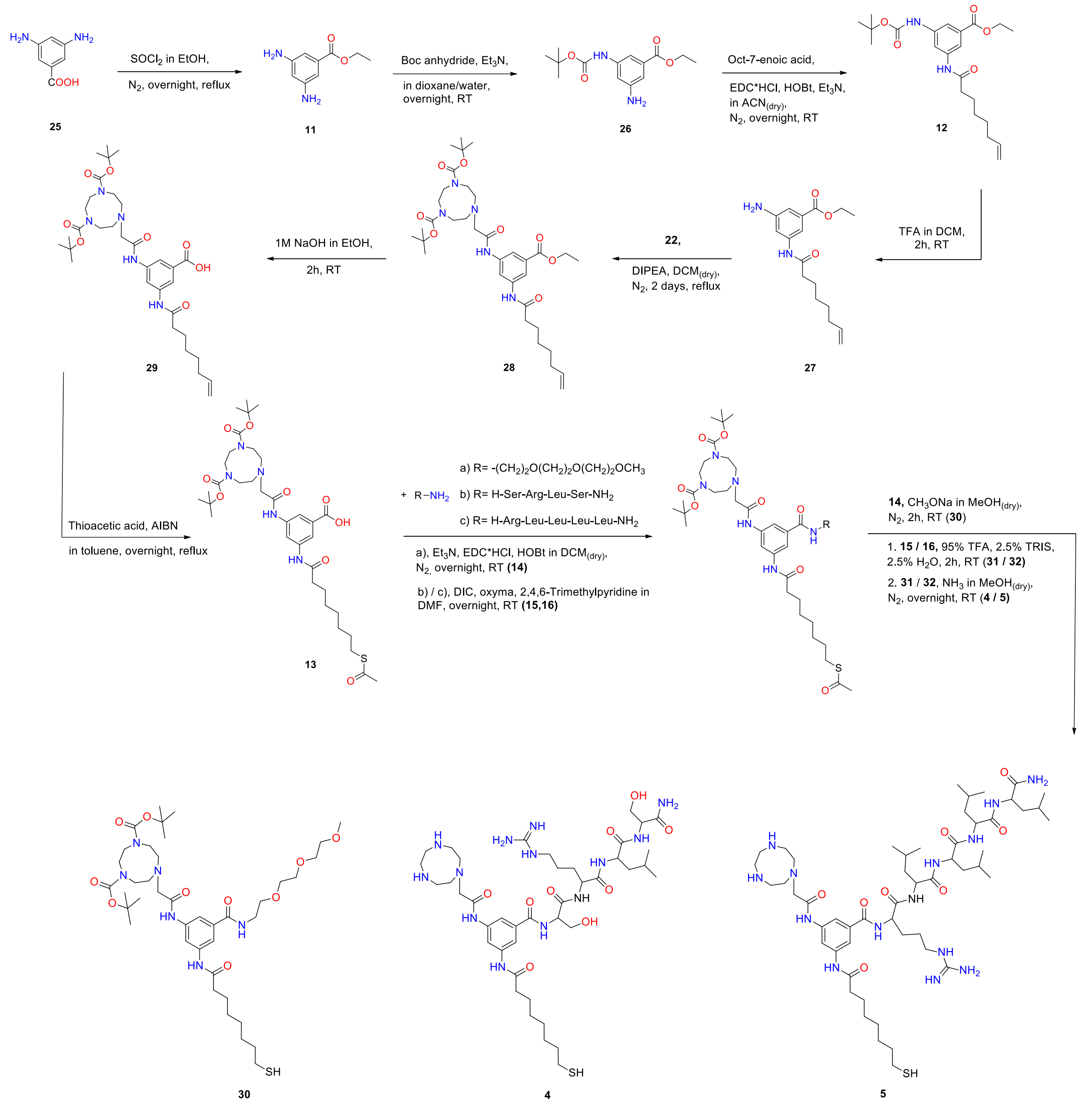

Ethyl 3,5-diaminobenzoate (11): In a two-neck round bottom flask, thionyl chloride ($SOCl_2$; 1.0 eq.) was added dropwise to dry ethanol under nitrogen at 0°C. Afterwards, **25** (3.0 eq.) was added in 3 portions and the resulting mixture was stirred at reflux overnight. The ethanol and unreacted $SOCl_2$ were evaporated, remaining residue was dissolved in water and alkalized with sat. aqueous solution of $NaHCO_3$. The cloudy mixture was extracted with ethyl acetate, collected organic phases were washed with water and dried over a $MgSO_4$. Brown crystals were obtained (91% yield).

^{1}H-NMR ($CDCl_3$, 500 MHz), δ: 6.79 (s, 2H), 6.18 (s, 1H), 4.31 (q, 2H), 3.66 (s, 4H), 1.36 (t, 3H)

ESI-MS (m/z): 181.0 $[M+H]^+$

Ethyl 3-amino-5-((tert-butoxycarbonyl)amino)benzoate (26): In a round-bottom flask placed in an ice bath, **11** (1.0 eq.) was solubilized in a mixture of dioxane and water (2:1), followed by addition of triethylamine (Et_3N; 2.2 eq.). Next, di-*tert*-butyl-dicarbonate ($(Boc)_2O$; 1.0 eq.) was solubilized in a small amount of dioxane and added dropwise to the mixture. The reaction was stirred at room temperature overnight. The solvents were evaporated and the crude was dissolved in ethyl acetate and washed with 10% aqueous solution of citric acid and water. The collected organic phases were dried over $MgSO_4$, concentrated in vacuo and purified by column chromatography (dichloromethane/acetonitrile 95/5-90/10). The desired product formed pale-yellow crystals (80% yield).

^{1}H-NMR ($CDCl_3$, 500 MHz), δ: 7.29 (s, 1H), 7.26 (s, 1H), 7.14 (s, 1H), 6.52 (s, 1H), 4.32 (q, 2H), 1.51 (s, 9H), 1.36 (t, 3H)

Ethyl 3-((tert-butoxycarbonyl)amino)-5-(oct-7-enamido)benzoate (12): In a two-neck round-bottom flask previously flushed with nitrogen, **26** (1.2 eq.) was dissolved in dry acetonitrile. Triethylamine (Et_3N; 3.0 eq.) hydroxybenzotriazole (3.0 eq.) and oct-7-enoic acid (1.0 eq.) were added. Afterwards the flask was cooled down in an ice-bath and after 15 minutes, 1-ethyl-3-(3-dimethylaminopropyl)carbodiimide chloride (EDC*HCl; 3.0 eq.) was added. The reaction was left stirring at room temperature for 24 hours. Then the mixture was concentrated in vacuo and purified by column chromatography (dichloromethane/acetonitrile 95/5-90/10). White solid was obtained (89% yield).

^{1}H-NMR ($CDCl_3$, 500 MHz), δ: 8.04 (s, 1H), 7.82 (s, 1H), 7.69 (s, 1H), 7.23 (s, 1H), 6.62 (s, 1H), 5.84-5.76 (m, 1H), 5.02-4.94 (m, 2H), 4.35 (q, 2H), 2.34 (t, 2H), 2.07 (q, 2H), 1.71 (m, 2H), 1.52 (s, 9H), 1.45-1.36 (m, 9H)
^{13}C-NMR ($CDCl_3$, 500 MHz), δ: 171.8, 166.5, 153.0, 139.6, 139.2, 132.2, 115.4, 114.9, 114.07, 81.41, 61.67, 38.12, 33.94, 29.07, 28.70, 25.72, 14.71

Ethyl 3-amino-5-(oct-7-enamido)benzoate (27): In a two-neck round-bottom flask previously flushed with nitrogen and placed in an ice bath, **12** (1.0 eq.) was solubilized in dry dichloromethane. Trifluoroacetic acid (TFA; 17 eq.) was dissolved in an equal amount of dichloromethane and added dropwise. Afterwards the reaction was stirred at room temperature for 2 hours. The solvent and TFA were evaporated and remaining oil was washed several times with cold diethyl ether. Desired product was isolated as a pale-yellow solid (82% yield).

^{1}H-NMR ($CDCl_3$, 500 MHz), δ: 7.60 (s, 1H), 7.32 (m, 2H), 7.15 (s, 1H), 5.83-5.75 (m, 1H), 5.01-4.93 (m, 2H), 4.34 (q, 2H), 2.95 (s, 2H), 2.36 (t, 2H), 2.05 (m, 2H), 1.71 (m, 2H), 1.38-1.25 (m, 9H)
^{13}C-NMR ($CDCl_3$, 500 MHz), δ: 171.6, 163.4, 147.0, 139.1, 138.8, 131.8, 114.5, 116.7, 110.7, 61.07, 37.73, 33.54, 28.67, 25.37, 14.29
ESI-MS (m/z): 305.2 $[M + H]^+$, 327.2 $[M + Na]^+$

Di-tert-butyl-7-(2-((3-(ethoxycarbonyl)-5-(oct-7-enamido)phenyl)amino)-2-oxoethyl)-1,4,7-triazonane-1,4 dicarboxylate (28): In a two-neck round-bottom flask previously purged with nitrogen, **27** (1.0 eq.) and **22** (1.1 eq.) were dissolved in dry dichloromethane. The flask was placed in

an ice-bath and after 15 minutes, DIPEA (5.0 eq.) was added dropwise. After the complete addition, the mixture was stirred and refluxed for 2 days. The solution was concentrated and purified by column chromatography (chloroform/acetone 92/8-90/10). The product was isolated as a white solid (56% yield).

^{1}H-NMR (CD_3OD, 500 MHz), δ: 8.20 (s, 1H), 8.01 (m, 2H), 5.87-5.79 (m, 1H), 5.03-4.95 (m, 2H), 4.37 (q, 2H), 3.56-3.70 (m, 10H), 2.91-2.82 (m, 4H), 2.41 (t, 2H), 2.10 (m, 2H), 1.72 (m, 2H), 1.50-1.38 (m, 25H)
^{13}C-NMR (CD_3OD, 500 MHz), δ: 174.5, 172.7, 167.4, 158.0, 157.6, 140.1, 139.9, 132.6, 118.6, 118.3, 117.9, 117.8, 114.93, 114.90, 81.55, 62.75, 62.30, 55.67, 55.36, 54.95, 51.86, 51.45, 37.91, 34.71, 29.80, 28.80, 26.68, 26.68, 14.62
ESI-MS (m/z): 674.4 $[M + H]^+$, 699.4 $[M + Na]^+$

3-(2-(4,7-bis(tert-butoxycarbonyl)-1,4,7-triazonan-1-yl)acetamido)-5-(oct-7-enamido)-benzoic acid (29): In a round-bottom flask placed in an ice-bath, **28** (1.0 eq.) was dissolved in ethanol. Next, an equal volume of aqueous 1M NaOH as ethanol was added dropwise. After the whole addition the cloudy mixture was stirred at room temperature for 2 hours. The solvent was evaporated, residue acidified with saturated aqueous solution of $KHSO_4$ (pH = 4). Afterwards the mixture was extracted with ethyl acetate, collected organic phases were washed with water and dried over a $MgSO_4$. Desired product was collected as a white solid (85% yield).

^{1}H-NMR ($CDCl_3$, 500 MHz), δ: 9.72 (m, 1H), 8.25-7.97 (m, 3H), 7.63 (s, 1H), 5.82-5.77 (m, 1H), 5.01-4.92 (m, 2H), 3.56-3.38 (m, 10H), 2.75 (m,4H), 2.37 (m, 2H), 2.06 (m, 2H), 1.72 (m, 2H), 1.48-1.41 (m, 22H)
^{13}C-NMR ($CDCl_3$, 500 MHz), δ: 174.6, 172.5, 169.0, 157.8, 157.3, 140.5, 139.7, 132.9, 118.5, 118.0, 117.6, 114.8, 81.42, 62.61, 55.51, 54.78, 53.33, 51.63, 51.13, 50.94, 37.75, 34.55, 29.64, 29.60, 28.63, 26.54
ESI-MS (m/z): 646.3 $[M + H]^+$, 668.3 $[M + Na]^+$

3-(8-(Acetylthio)octanamido)-5-(2-(4,7-bis(tert-butoxycarbonyl)-1,4,7-triazonan-1-yl)acetamido)benzoic acid (13): In a round-bottom flask, **29** (1.0 eq.) and *α,α'*-azoisobutyronitrile (AIBN; 0.5 eq.) were dissolved in toluene. Next, thioacetic acid (3.5 eq.) was added and the reaction mixture was refluxed overnight. The toluene was evaporated and the remaining oily crude was dried under high vacuum and purified by column chromatography (dichloromethane/methanol 90/10-80/20 with 0.5% addition of TEA). The product was obtained as a pale-yellow solid (80% yield).

^{1}H-NMR (CD_3OD, 500 MHz), δ: 8.17 (m, 1H), 7.95-7.91 (m, 2H), 3.55-3.32 (m, 10H), 2.87-2.81 (m, 6H), 2.38 (t, 2H), 2.30 (s, 3H), 1.70 (m, 2H), 1.57 (m, 2H), 1.48-1.26 (m, 24H)
^{13}C-NMR (CD_3OD, 500 MHz), δ: 197.6, 174.7, 172.5, 157.9, 157.5, 140.2, 139.5, 118.7, 118.2, 117.1, 81.41, 81.34, 62.54, 55.63, 55.35, 54.94, 51.76, 51.50, 51.16, 37.94, 30.70, 30.51, 30.13, 29.90, 29.81, 29.63, 28.81, 26.81
ESI-MS (m/z): 722.4 $[M + H]^+$, 744.4 $[M + Na]^+$

Di-tert-butyl-7-(2-((3-(8-(acetylthio)octanamido)-5-((2-(2-(2-methoxyethoxy)ethoxy)ethyl)-carbamoyl)-phenyl)-amino)-2-oxoethyl)-1,4,7-triazonane-1,4-dicarboxylate (14): In a two-neck round-bottom flask previously flushed with nitrogen, **13** (1.0 eq.) was dissolved in dry acetonitrile.

Triethylamine (Et_3N; 3.0 eq.), hydroxybenzotriazole (HOBt; 3.0 eq.) and 2-(2-(2-methoxyethoxy)ethoxy)ethan-1-amine (**(a)**, 1.2 eq.) were added. The flask was placed in an ice-bath and when the mixture reached 0°C, 1-ethyl-3-(3-dimethylaminopropyl)carbodiimide (EDC*HCl; 3.0 eq.) was added. The reaction was stirred at room temperature under nitrogen overnight. Then the crude was concentrated and purified by column chromatography (dichloromethane/methanol 96/4). The product was isolated as a pale-yellow solid (71% yield).

^{1}H-NMR ($CDCl_3$, 500 MHz), δ: 8.35-7.64 (m, 3H), 3.68-3.34 (m, 25H), 2.87-2.75 (m, 6H), 2.38-2.32 (m, 5H), 1.69 (m, 2H), 1.56 (m, 2H), 1.48-1.25 (m, 24H)

^{13}C-NMR ($CDCl_3$, 500 MHz), δ: 196.3, 188.2, 171.8, 167.3, 156.6, 156.0, 139.5, 139.0, 135.9, 115.1, 114.9, 114.5, 80.61, 80.42, 71.95, 70.63, 70.55, 70.37, 69.95, 63.70, 59.04, 55.20, 54.21, 53.13, 51.42, 50.21, 49.42, 40.03, 37.63, 30.77, 29.54, 29.16, 28.93, 28.65, 25.52

ESI-MS (m/z): 867.6 $[M + H]^+$

Di-*tert*-butyl 7-(2-((3-(8-(acetylthio)octanamido)-5-((1-((1-((1-((1-amino-3-hydroxy-1-oxopropan-2-yl)amino)-4-methyl-1-oxopentan-2-yl)amino)-5-guanidino-1-oxopentan-2-yl)amino)-3-hydroxy-1-oxopropan-2-yl)carbamoyl)phenyl)amino)-2-oxoethyl)-1,4,7-triazonane-1,4-dicarboxylate and di-*tert*-butyl 7-(2-((3-(8-(acetylthio)octanamido)-5-((1-amino-15-carbamoyl-1-imino-9,12-diisobutyl-17-methyl-7,10,13-trioxo-2,8,11,14-tetraazaoctadecan-6-yl)carbamoyl)phenyl)amino)-2-oxoethyl)-1,4,7-triazonane-1,4-dicarboxylate, (15) and (16): In a two-neck round-bottom flask previously flushed with nitrogen, **13** (1.0 eq.) together with collidine (2,4,6-trimethylpyridine, 3.0 eq.) and N,N'-diisopropylcarbodiimide (DIC; 3.0 eq.) were dissolved in dry DMF. Then, the solution of previously synthesized peptide **(b)** or **(c)** ((the synthesis shown below), 1.2 eq.) with oxyma (ethyl cyano(9ydroxyamino)acetate, 3.0 eq.) in dry DMF were added. The mixture was stirred under nitrogen overnight. The solvent was evaporated and the crude purified by preparative RP-HPLC (45 min. run, 0-60% + 0.05% TFA, λ=214 nm). The collected fractions were lyophilized yielding a white solid.

*LC-MS (m/z): **15:** 582.68 $[0.5M + H]^+$, 1164.52 $[M + H]^+$*

*LC-MS (m/z): **16:** 665.43 $[0.5M + H]^+$, 1329.62 $[M + H]^+$*

Di-tert-butyl-7-(2-((3-(8-mercaptooctanamido)-5-((2-(2-(2-methoxyethoxy)ethoxy)ethyl)-carbamoyl)phenyl)-amino)-2-oxoethyl)-1,4,7-triazonane-1,4-dicarboxylate (30): In a two-neck round-bottom flask previously flushed with nitrogen, **14** (0.1 mmol) was solubilized in a small amount of dry methanol. Sodium methoxide (2.0 eq.) was added. The mixture was stirred at RT under nitrogen for 2 hours. Next methanol was evaporated and the crude was dried under high vacuum. The obtained product, being a precursor of thiol **3**, was used *in situ* without any additional purification in the synthesis of gold nanoparticles.

Once AuNPs were formed and purified, Boc-protecting groups were removed by dissolving AuNPs in dry dichloromethane and slow addition trifluoroacetic acid in DCM (until gold nanoparticles started precipitating). The mixture was stirred for 2 hours, solvent and acid were evaporated and gold nanoparticles were purified again on a 25-Sephadex column, using MeOH as an eluent (thiol **3**, **AuNP3**).

7-(2-((3-(8-(acetylthio)octanamido)-5-((1-((5-((amino(iminio)methyl)amino)-1-((1-((1-amino-3-hydroxy-1-oxopropan-2-yl)amino)-4-methyl-1-oxopentan-2-yl)amino)-1-oxopentan-2-yl)amino)-3-hydroxy-1-oxopropan-2-yl)carbamoyl)phenyl)amino)-2-oxoethyl)-1,4,7-triazonane-1,4-diium and 7-(2-((3-(8-(acetylthio)octanamido)-5-((1-amino-15-carbamoyl-1-iminio-9,12-diisobutyl-17-methyl-7,10,13-trioxo-2,8,11,14-tetraazaoctadecan-6-yl)carbamoyl)phenyl)amino)-2-oxoethyl)-1,4,7-triazonane-1,4-diium (31) and (32): In a two-neck round-bottom flask previously flushed with nitrogen and placed in an ice bath, **15** / **16** (1.0 eq.) was solubilized in dry dichloromethane. Trifluoroacetic acid (TFA; 17 eq.) was dissolved in an equal amount of dichloromethane and added dropwise. Afterwards the reaction was stirred at room temperature for 4 hours. The solvent and TFA were evaporated and remaining yellow oil was precipitated from a cold *tert*-buthylmethyl ether. Both compounds were obtained as a white solid (54-62%).

Next, the solution of ammonia in methanol (2.0 eq.) was added and the mixture was stirred at room temperature overnight (the reaction was monitored by ^{1}H NMR). The solvents were evaporated and the crude was dried under high vacuum. Without any additional steps the product with a free thiol group was used *in situ* in the synthesis of gold nanoparticles (thiols **4**,**5**; **AuNP4,5**).

4. The solid-phase synthesis of peptides

H-Ser-Arg-Leu-Ser-NH_2 and H-Arg-Leu-Leu-Leu-Leu-NH_2 (b) and (c): In a 25 mL volume syringe equipped with a filter, 1.0 g of the resin (TentaGel S RAM) was added. It was swelled in dichloromethane for 30 minutes. Afterwards the solvent was filtered off and the Fmoc groups were deprotected with 20% solution of piperidine in *N,N*-dimethylformamide (15-minute stirring). Next, all liquids were filtrated and the resin was washed with 1-methyl-2-pyrrolidone (NMP), methanol and dichloromethane. Simultaneously the protected amino acid (amounts depicted in the table below), oxyma (ethyl cyano(11ydroxyamino)acetate; 1.0 eq.), *N,N'*-diisopropylcarbodiimide (DIC; 1.0 eq.) and collidine (2,4,6-Trimethylpyridine; 1.0 eq.) were dissolved in *N*-methyl-2-pyrrolidon and sonicated. Resulting mixture was poured on the resin.

Compound	MW [g/mol]	Quantity [mg]	mmol	Equivalents
Fmoc-Ser(tBu)-OH	383.4	479.3	1.25	1
Fmoc-Leu-OH	353.5	441.8	1.25	1
Fmoc-Arg(Pbf)-OH	648.7	810.9	1.25	1

A total volume was adjusted using dichloromethane (up to 15 mL) and then everything was stirred for 1 hour. Afterwards the solvents were filtrated, resin washed with NMP, MeOH and DCM. The Fmoc group of the last coupled amino acid was deprotected by 20% solution of piperidine in DMF. The liquids were again filtered off and the resin washed with NMP, MeOH and DCM. Described steps were repeated until desired oligopeptide was built. After the last coupling, the cocktail made from 95% (v) of TFA, 2.5% (v) of triisopropylsilane and 2.5% (v) of water was poured into dry resin, in order to cleave the oligopeptide from the solid platform. The concoction was stirred for 2 hours. Next the crude was quenched and washed several times with cold *tert*-butylmethyl ether. The protocol yielded a formation of desired compounds with high purity.

LC-MS (m/z): ***b)*** *461.41 $[M+H]^+$*
LC-MS (m/z): ***c)*** *626.50 $[M+H]^+$*

5. The synthesis of 2-hydroxypropyl *p*-nitrophenyl phosphate (34)

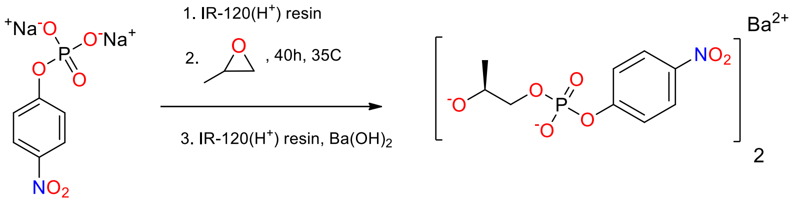

33 **34**

The synthesis of HPNP was performed basing on the method published by Brown and Usher.[1]

33 (5.0 mmol) was dissolved in 10 mL of distilled water. The solution was passed through an ion-exchange column (IR-120 (H+) resin). The acid effluent was alkalized to pH 8.0 with an aqueous solution of ammonia. Following this, 1,2-epoxypropane (17.0 mmol) was added and the reaction was stirred at 40°C for 2 days and any unreacted epoxide was removed in vacuo. The remaining liquid was diluted with a small amount of water and passed through an ion-exchange column ((IR-120 (H+) resin). The aqueous solution was carefully neutralized to pH 6.5-7.0 with an aqueous solution of barium hydroxide (carbonate-free) and then concentrated in vacuo at room temperature until the volume was reduced to 70%. Ethanol (2 vol.) was added and the white precipitate was filtered off. The filtrate was concentrated and added to vigorously stirring 10% solution of ethanol in acetone (300 ml). The mixture was stirred for additional 1 hour. After that time a white precipitate was filtered off and dried under high pressure. Desired compound was obtained as a white fluffy solid.
(46% yield).

^{1}H-NMR (D_2O, 500 MHz), δ: 8.26 (d, 2H), 7.36 (d, 2H), 4.02 (d, 2H), 3.95 (m, 1H), 1.15 (d, 3H)
^{13}C-NMR (D_2O, 500 MHz), δ: 157.3, 143.4, 125.8, 120.4, 71.00, 66.48, 17.64
ESI-MS (m/z): 275.8 $[M]^-$
^{31}P-NMR (D_2O, 500 MHz), δ: -4.83, -4.86, -4.89
Elemental Analysis ($C_{18}H_{22}BaN_2O_{14}P_2$): Called- C: 31.35, H: 3.22, N: 4.06; Found- C: 30.95, H: 3.23, N: 3.37

[1] Brown D.M., Usher D.A., *J. Chem. Soc.*, **1965**, 0, 6558-6564.

6. The synthesis of gold nanoparticles

The synthesis of AuNPs was performed using the method published by Scrimin.[2]

All the glassware was washed with Aqua Regia and rinsed with distilled water. The aqueous solution of $HauCl_4{\cdot}3H_2O$ (1.0 eq.) was extracted with tetraoctylammonium bromide (3.0 eq.) previously dissolved in degassed toluene. To the red-orange organic phase, dioctylamine (20 eq.) was added. The mixture was vigorously stirred at room temperature under nitrogen for 30 minutes. During that time the colour changed from an orange to colourless. The flask was placed in an ice-bath and then aqueous solution of $NaBH_4$ (10 eq.) was rapidly added. The colour changed immediately to brownish-black due to nanoparticles' formation. After 2 hr stirring, a small amount of left water was removed. Keeping the reaction under an ice-bath, desired thiol (2.0 eq.) dissolved in methanol was added to the solution and then the reaction was left stirring for additional 2 hours. AuNPs were purified by their sonication and washing with different solvents (hexane, toluene, petroleum ether, ethyl acetate and methanol). Eventually, their concentrated solution was applied on the gel permeation chromatography with Sephadex G-25 resin.

[2]Manea, F., Bindoli, C., Polizzi, S., Lay, L., Scrimin, P., *Langmuir,* **2008**, 24, 4120-4124.

7. Characterization of AuNP1

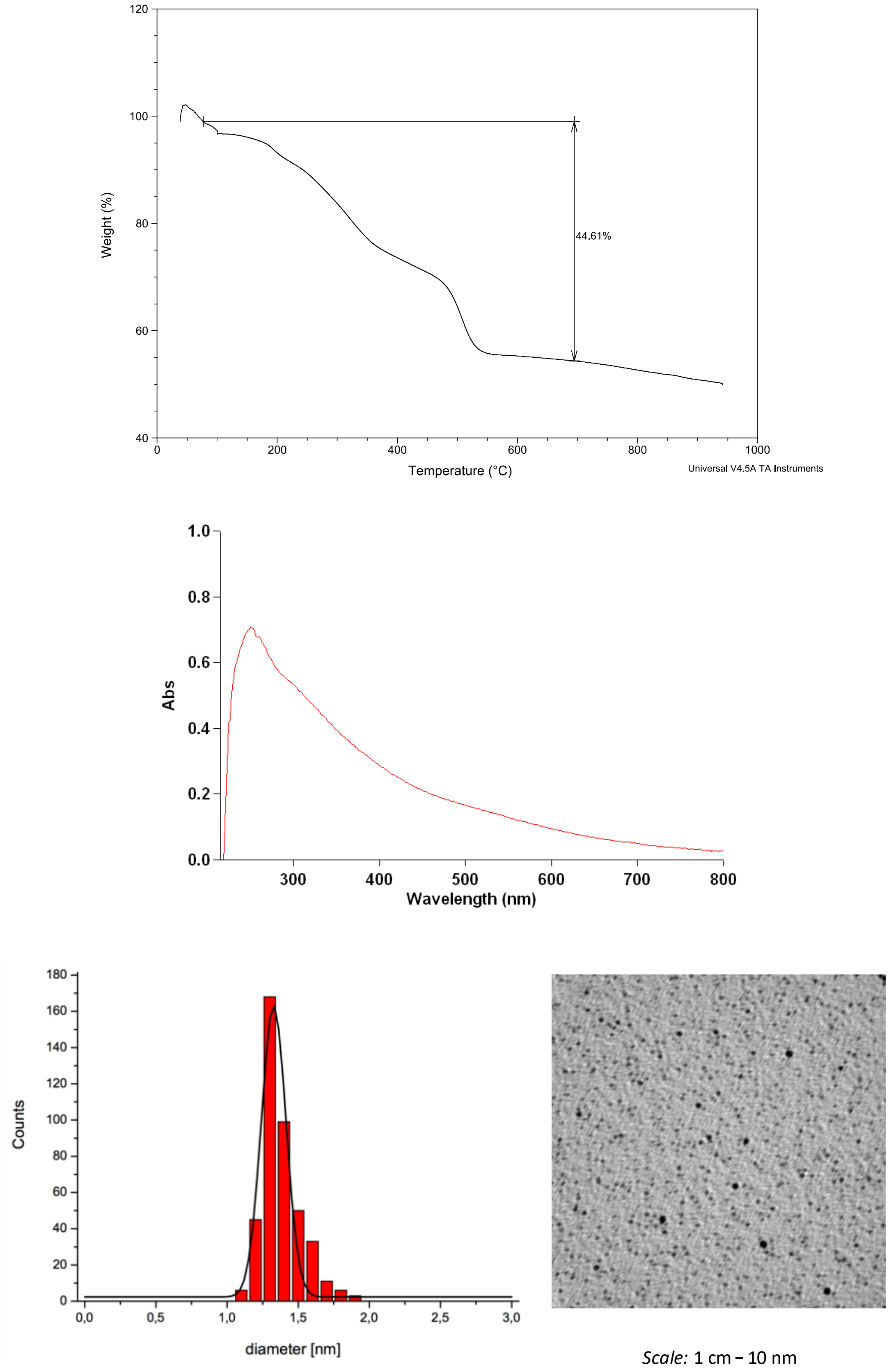

Figure S1. *Top*: TGA analysis of **AuNP1**.

Middle: UV-VIS analysis of **AuNP1.**

Bottom: TEM image and size distribution of **AuNP1**. Average diameter = 1.3 nm (± 0.2 nm).

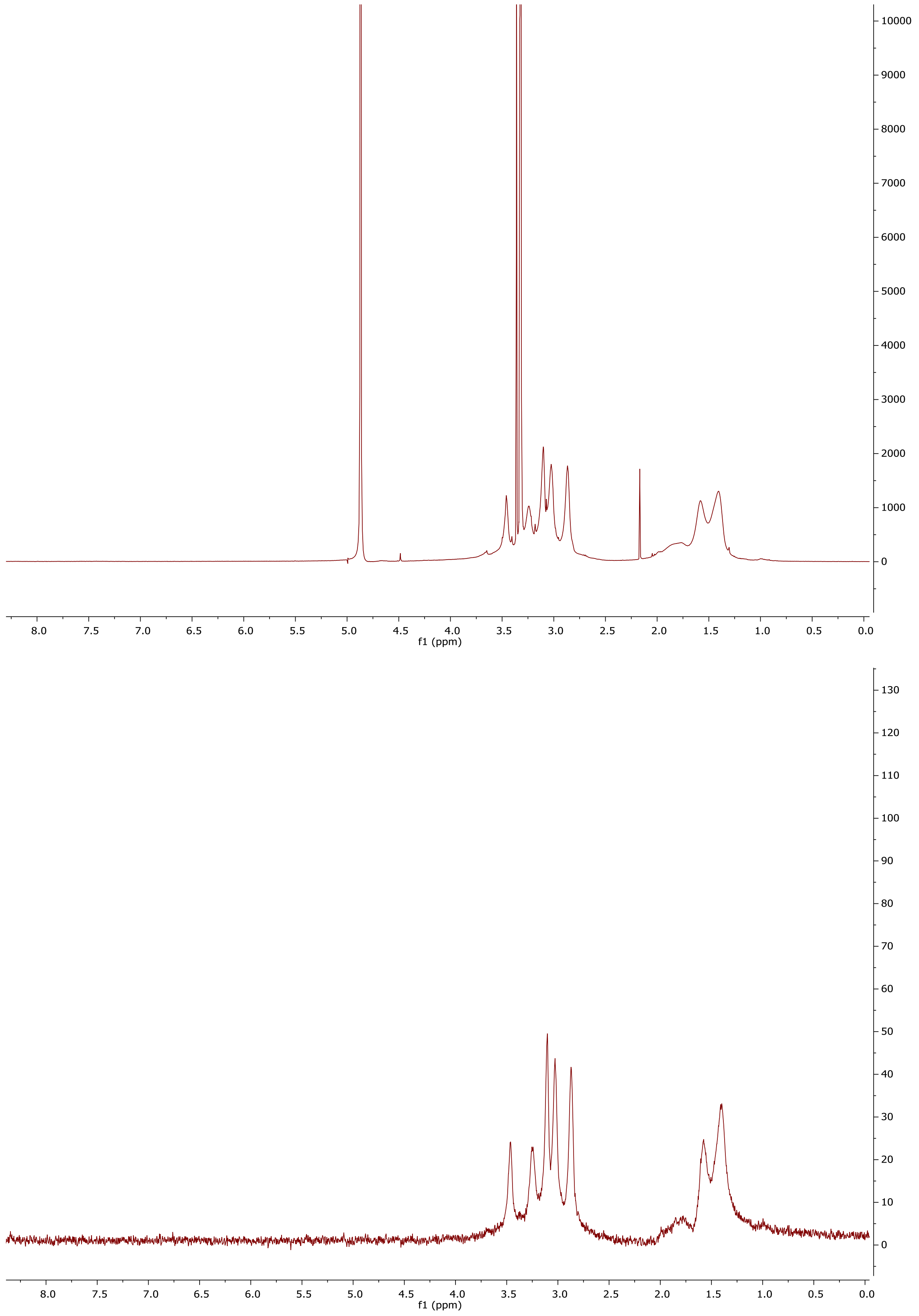

Figure S2. *Top:* ^{1}H-NMR (500 MHz) spectrum of **AuNP1** in CD_3OD.
Bottom: Diffusion-filtered ^{1}H-NMR (500 MHz) spectrum of **AuNP1** in CD_3OD.

8. Characterization of AuNP2

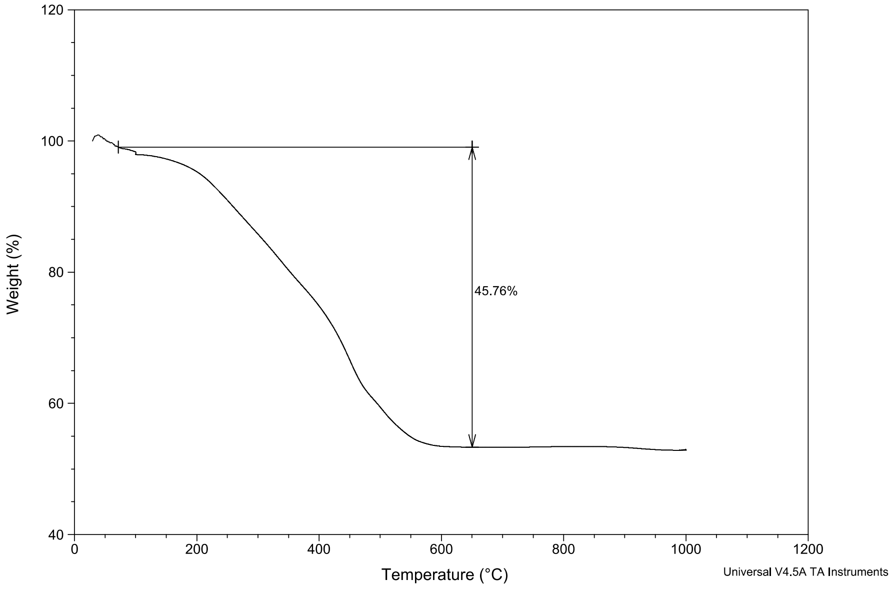

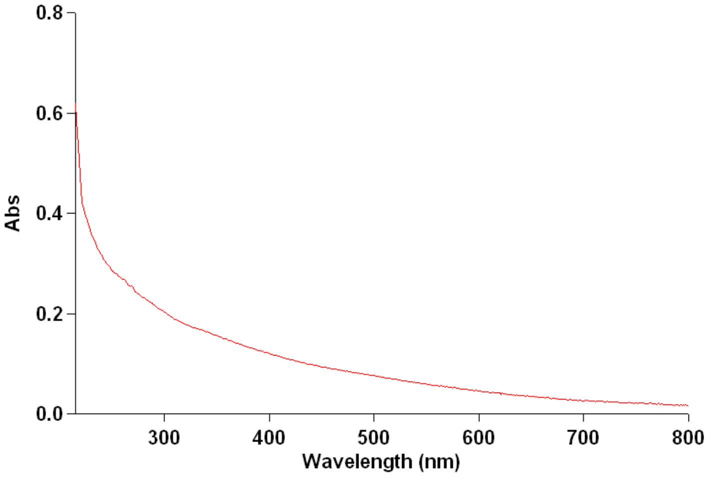

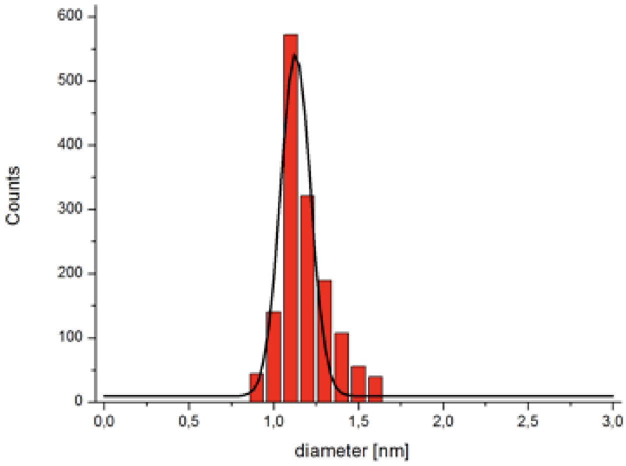

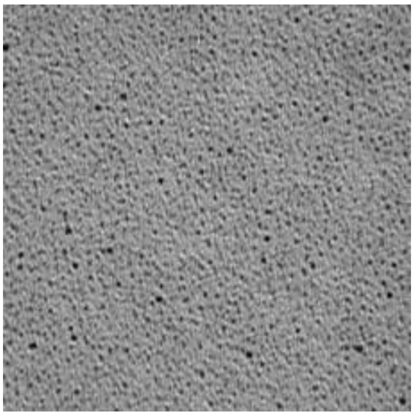

Scale: 1 cm – 20 nm

Figure S3. *Top*: TGA analysis of **AuNP2.**

Middle: UV-VIS analysis of **AuNP2.**

Bottom: TEM image and size distribution of **AuNP2.** Average diameter = 1.1 nm (± 0.2 nm)

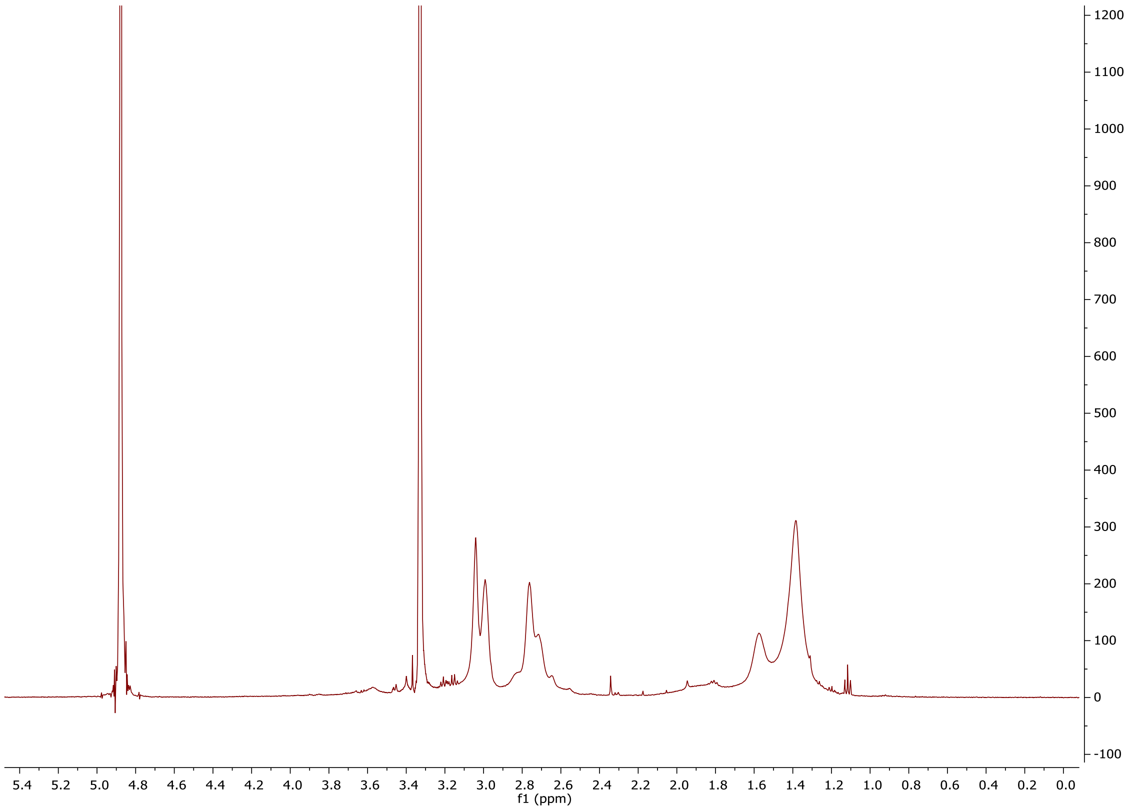

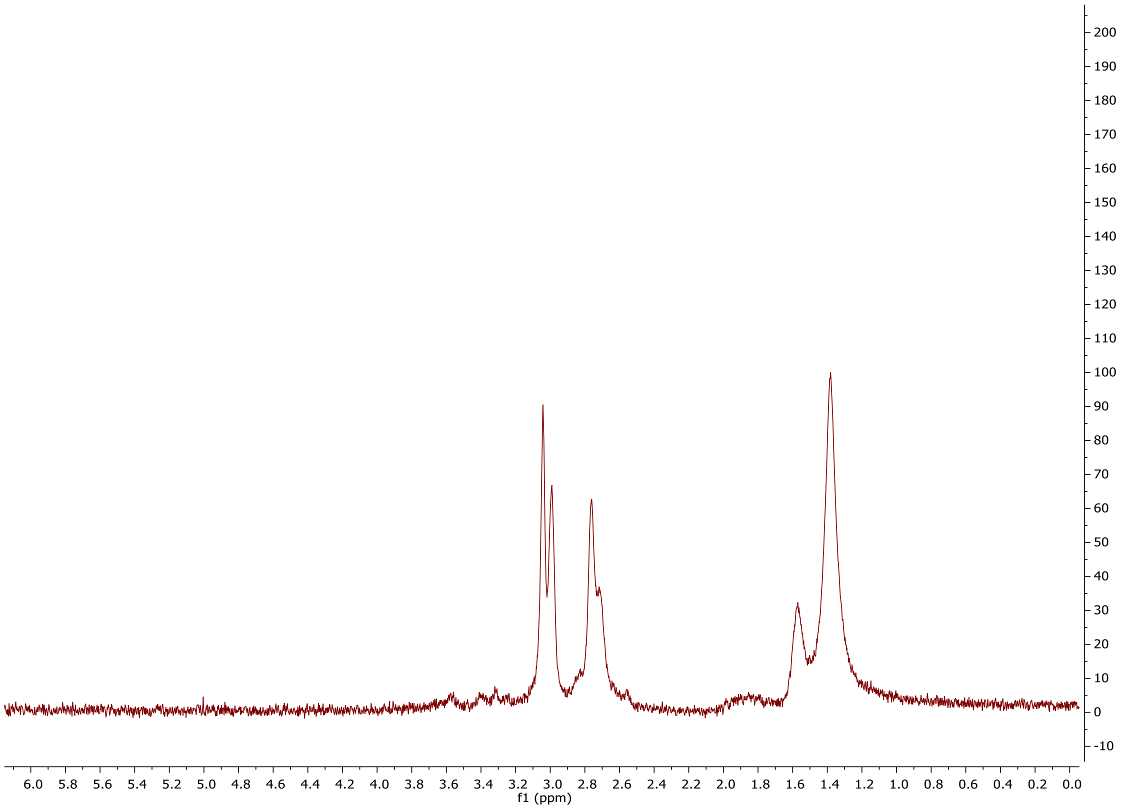

Figure S4. *Top:* ^{1}H-NMR (500 MHz) spectrum of **AuNP2** in CD_3OD.
Bottom: Diffusion-filtered ^{1}H-NMR (500 MHz) spectrum of **AuNP2** in CD_3OD.

9. Characterization of AuNP3

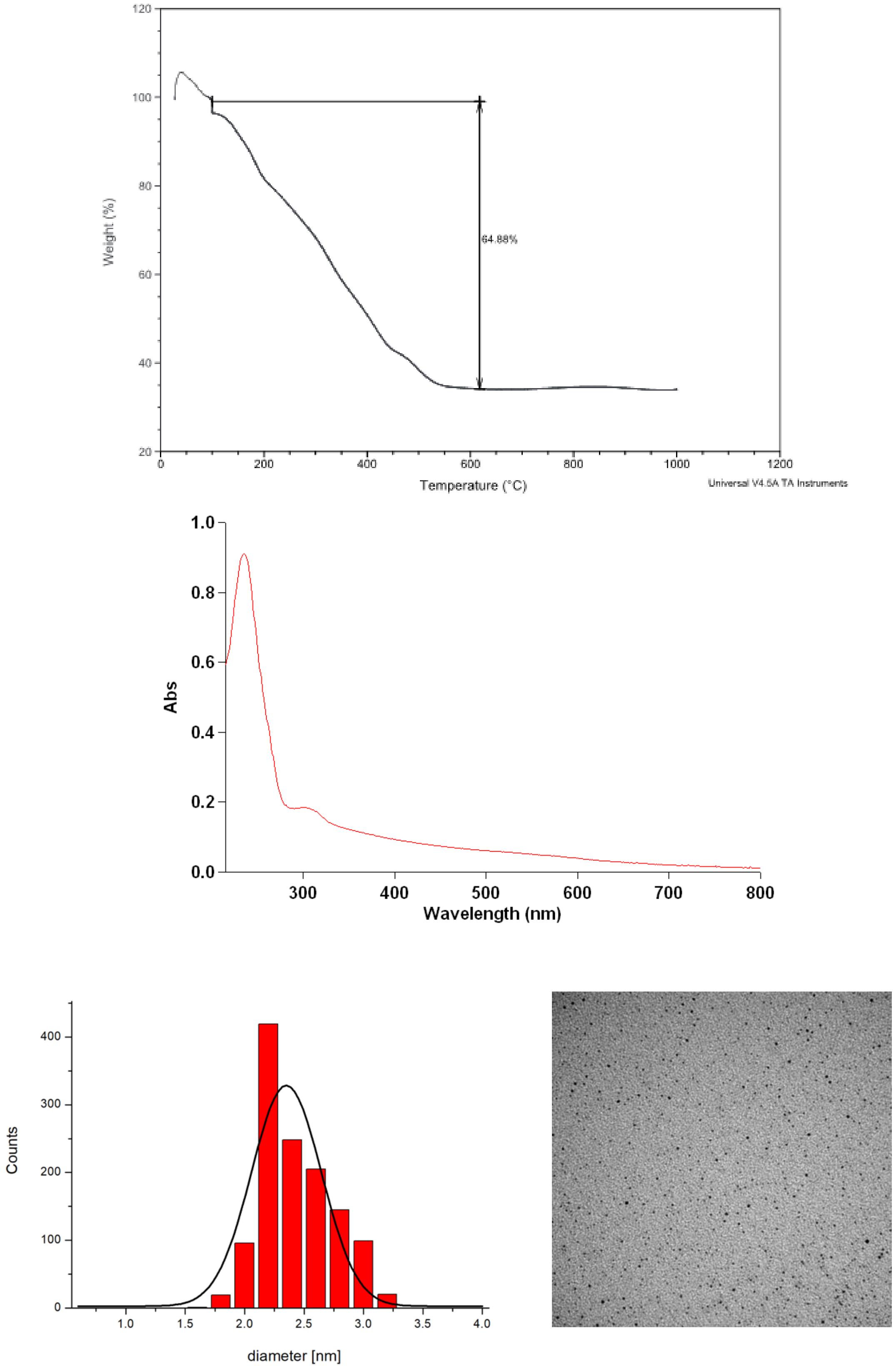

Scale: 1 cm – 50 nm

Figure S5. *Top*: TGA analysis of **AuNP3**.

Middle: UV-VIS analysis of **AuNP3**.

Bottom: TEM image and size distribution of **AuNP3**. Average diameter = 2.3 nm (± 0.3 nm).

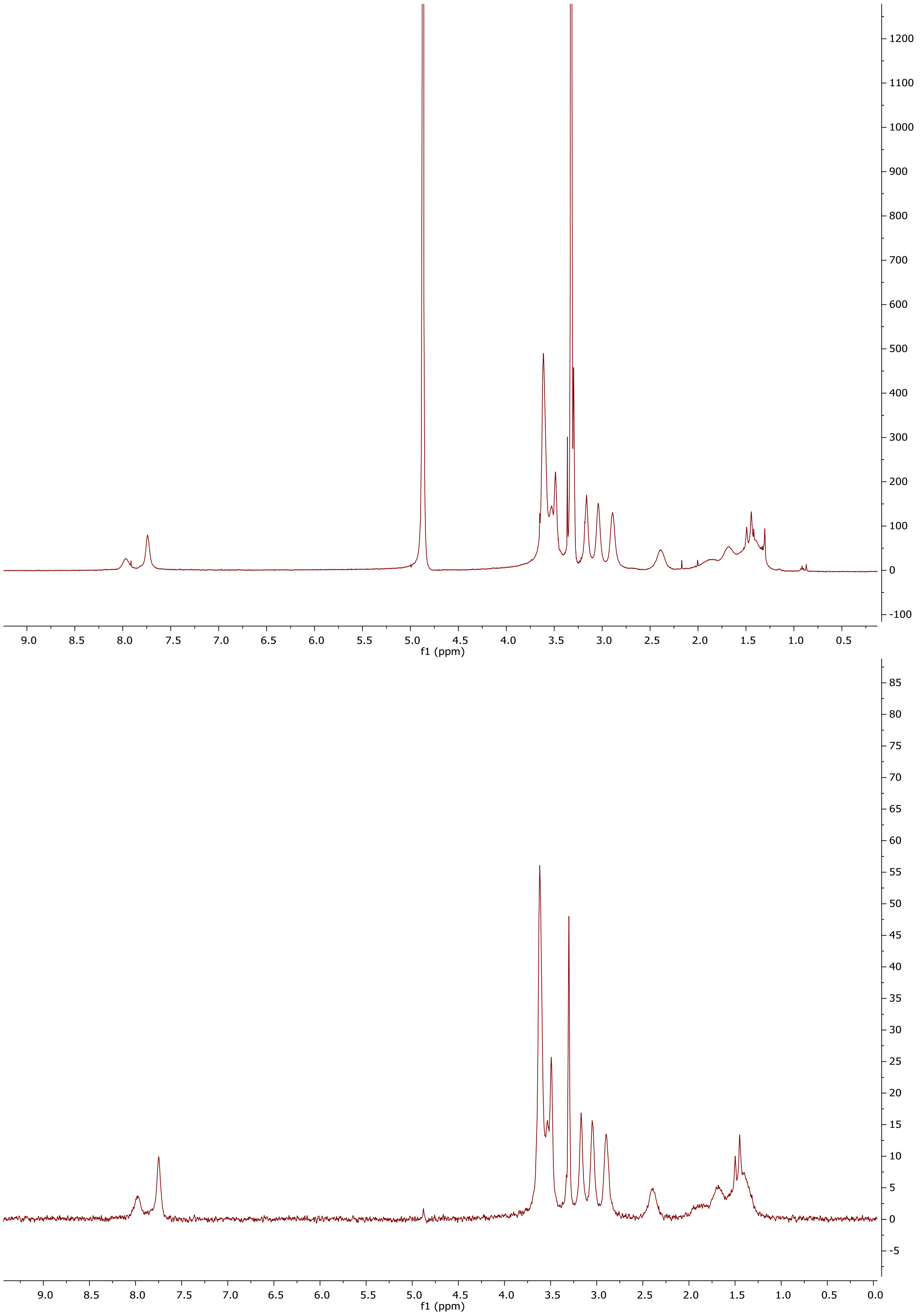

Figure S6. *Top:* ^{1}H-NMR (500 MHz) spectrum of **AuNP3** in CD_3OD.
Bottom: Diffusion-filtered ^{1}H-NMR (500 MHz) spectrum of **AuNP3** in CD_3OD.

10. Characterization of AuNP4

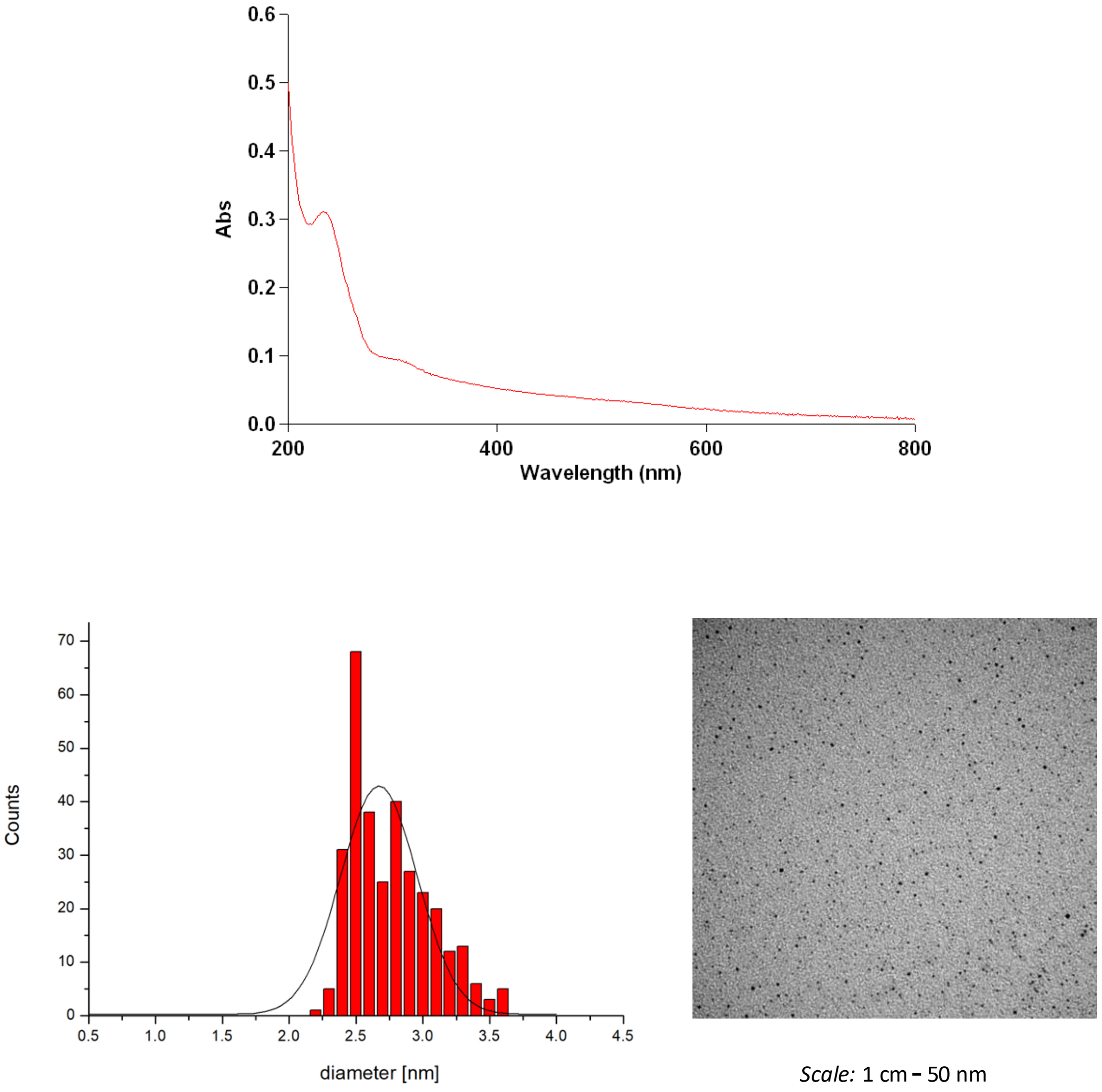

Figure S7. *Top*: UV-VIS analysis of **AuNP4.**
Bottom: TEM image and size distribution of **AuNP4**. Average diameter = 2.6 nm (± 0.3 nm).

*TGA analysis of **AuNP4** was not studied due to a very small amount of obtained nanoparticles.

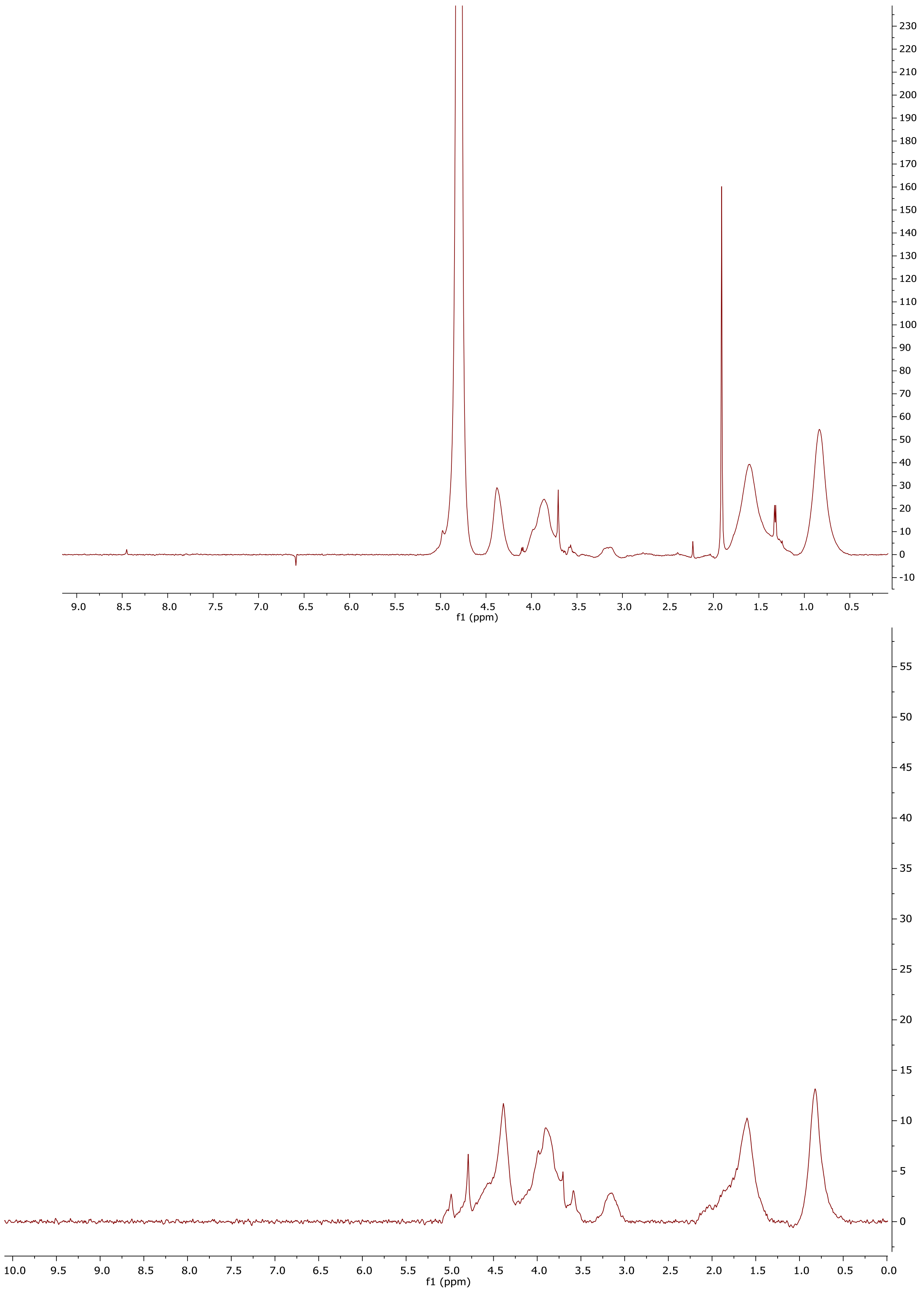

Figure S8. *Top:* ^{1}H-NMR (500 MHz) spectrum of **AuNP4** in D_2O.
Bottom: Diffusion-filtered ^{1}H-NMR (500 MHz) spectrum of **AuNP4** in D_2O.

11. Characterization of AuNP5

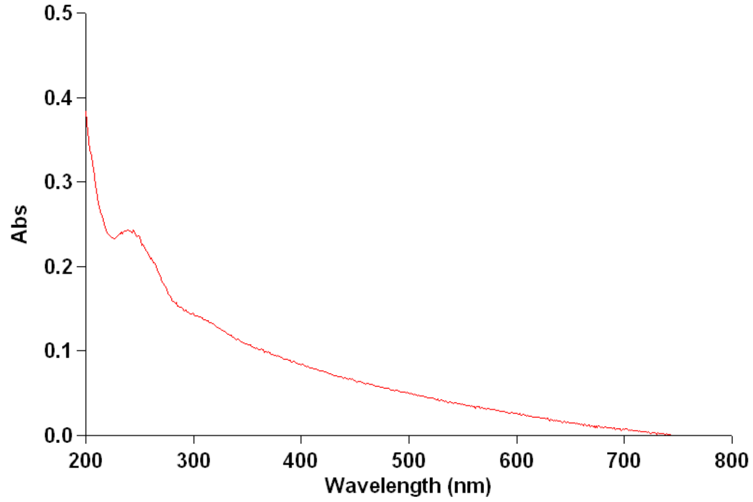

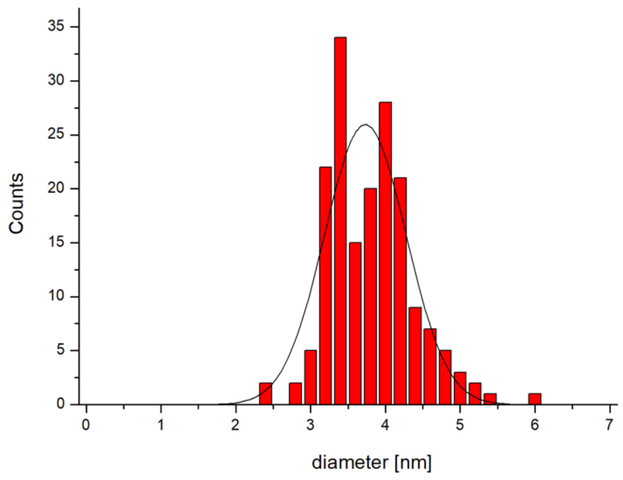

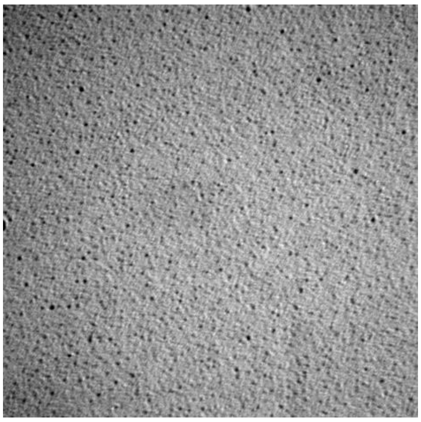

Scale: 1 cm – 50 nm

Figure S9. *Top*: UV-VIS analysis of **AuNP5.**

Bottom: TEM image and size distribution of **AuNP5**. Average diameter = 3.7 nm (± 0.5 nm).

*TGA analysis of **AuNP5** was not studied due to very small amount of obtained nanoparticles.

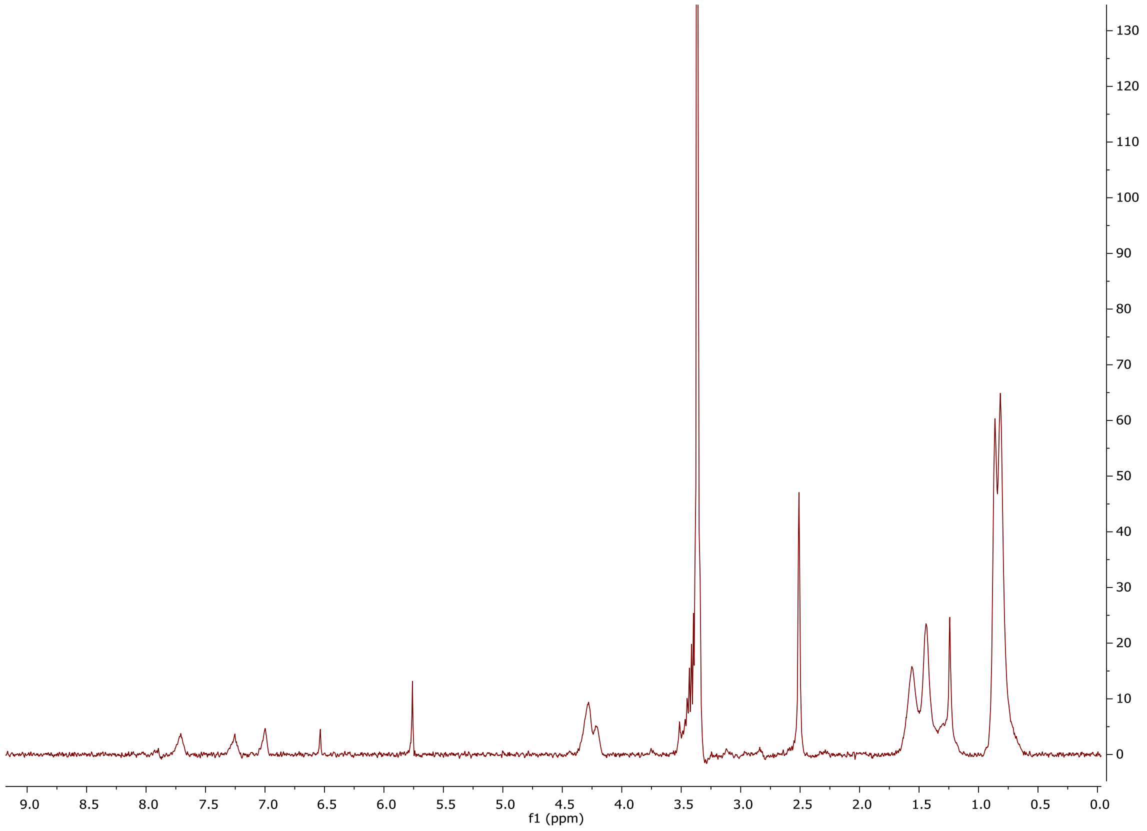

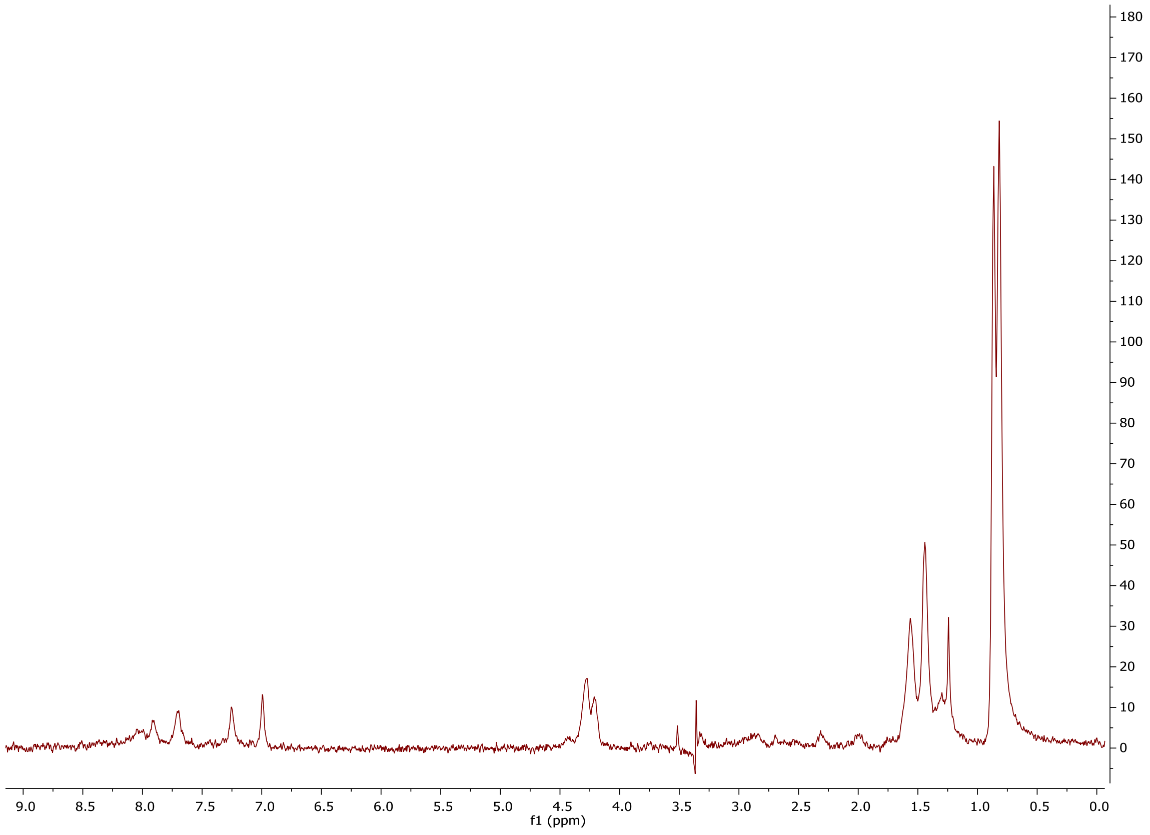

Figure S10. *Top:* ^{1}H-NMR (500 MHz) spectrum of **AuNP5** in d-DMSO.
Bottom: Diffusion-filtered ^{1}H-NMR (500 MHz) spectrum of **AuNP5** in d-DMSO.

12. pH vs pD profiles for the cleavage of HPNP by AuNP1

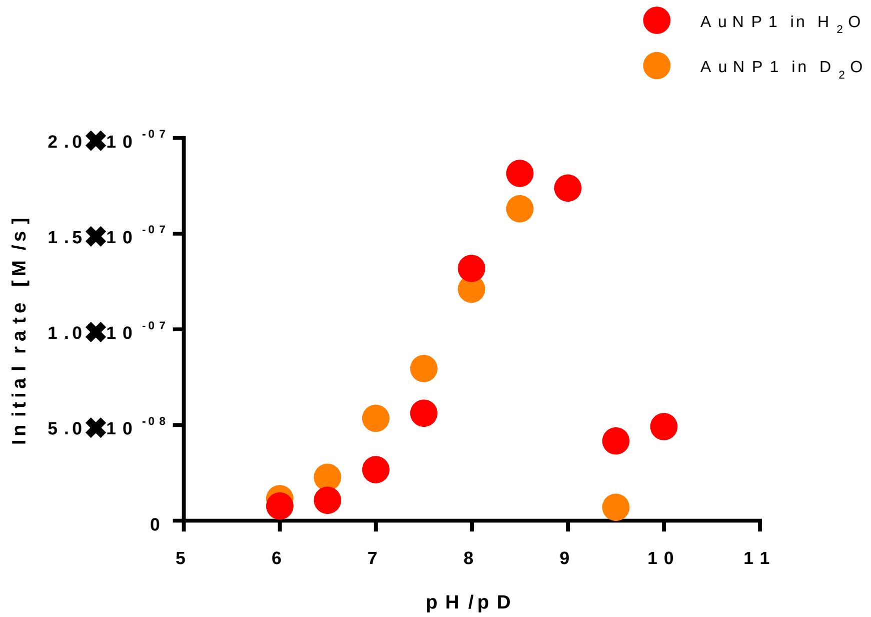

Figure S11. pH/pD rate profile of the cleavage of HPNP by **AuNP1** and Zn(II). Conditions: [**AuNP1**, in thiol units] = [Zn(II)] = 1.703 × 10^{-5} M, [buffers] = 1.0 × 10^{-2} M, [HPNP] = 1.17 × 10^{-3} M, 25 °C in H_2O/D_2O.
